# Photoredox-HAT Catalysis for Primary Amine α-C–H Alkylation: Mechanistic Insight with Transient Absorption Spectroscopy

**DOI:** 10.1021/acscatal.3c01474

**Published:** 2023-05-30

**Authors:** Mahima Sneha, Georgia L. Thornton, Luke Lewis-Borrell, Alison S. H. Ryder, Samuel G. Espley, Ian P. Clark, Alexander J. Cresswell, Matthew N. Grayson, Andrew J. Orr-Ewing

**Affiliations:** † School of Chemistry, University of Bristol, Cantock’s Close, Bristol BS8 1TS, U.K.; ‡ Department of Chemistry, Dartmouth College, Hanover, New Hampshire 03755, United States; § Centre for Sustainable Chemical Technologies, University of Bath, 1 South, Claverton Down, Bath BA2 7AY, U.K.; ∥ Department of Chemistry, University of Bath, 1 South, Claverton Down, Bath BA2 7AY, U.K.; ⊥ Central Laser Facility, Research Complex at Harwell, Science and Technology Facilities Council, Rutherford Appleton Laboratory, Harwell Oxford, Didcot OX11 0QX, U.K.

**Keywords:** photoredox catalysis, hydrogen-atom transfer, transient absorption spectroscopy, azidyl radical, organic photocatalyst

## Abstract

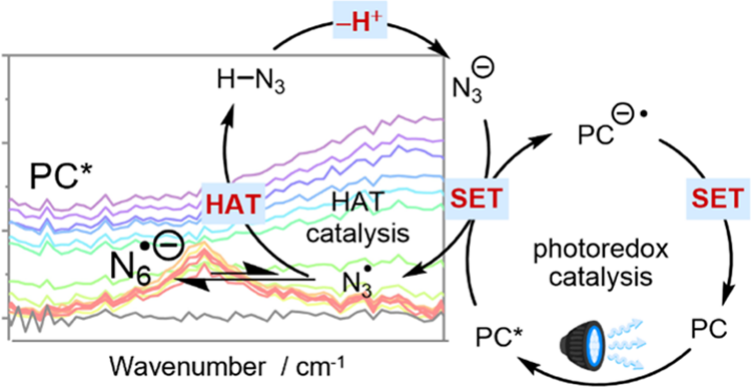

The synergistic use of (organo)photoredox catalysts with hydrogen-atom transfer (HAT) cocatalysts has emerged as a powerful strategy for innate C(sp^3^)–H bond functionalization, particularly for C–H bonds α- to nitrogen. Azide ion (N_3_^–^) was recently identified as an effective HAT catalyst for the challenging α-C–H alkylation of unprotected, primary alkylamines, in combination with dicyanoarene photocatalysts such as 1,2,3,5-tetrakis(carbazol-9-yl)-4,6-dicyanobenzene (4CzIPN). Here, time-resolved transient absorption spectroscopy over sub-picosecond to microsecond timescales provides kinetic and mechanistic details of the photoredox catalytic cycle in acetonitrile solution. Direct observation of the electron transfer from N_3_^–^ to photoexcited 4CzIPN reveals the participation of the S_1_ excited electronic state of the organic photocatalyst as an electron acceptor, but the N_3_^•^ radical product of this reaction is not observed. Instead, both time-resolved infrared and UV–visible spectroscopic measurements implicate rapid association of N_3_^•^ with N_3_^–^ (a favorable process in acetonitrile) to form the N_6_^•–^ radical anion. Electronic structure calculations indicate that N_3_^•^ is the active participant in the HAT reaction, suggesting a role for N_6_^•–^ as a reservoir that regulates the concentration of N_3_^•^.

## 1 Introduction

**Figure 1:**
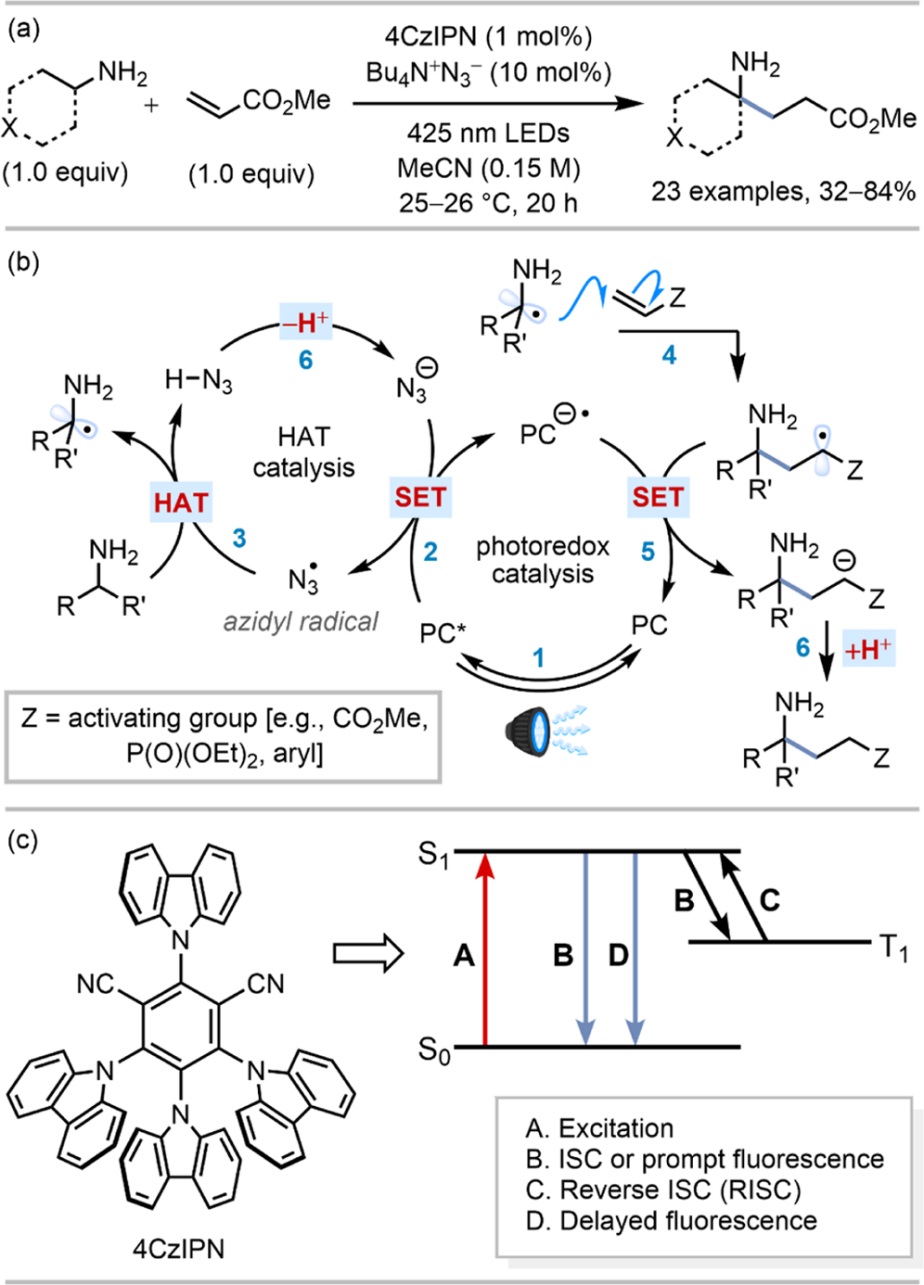
α-C–H alkylation of primary amines with various radical acceptors using photoredox-HAT catalysis with azide ion as the HAT catalyst. (a) General reaction scheme. (b) Proposed mechanism; the numbered steps are: 1—photoexcitation of the 4CzIPN photocatalyst (PC), with PC* denoting a photoexcited species in either its S_1_ or T_1_ state; 2—single electron transfer (SET) from azide ion to PC* forming a reduced PC radical anion, PC^•–^, and an azidyl radical, N_3_^•^; 3—HAT from a primary amine to the azidyl radical; 4—reaction of the α-amino radical with an alkene acceptor; 5—SET from PC^•–^ to the carbon-centered radical to produce a transient carbanion and recover the PC in its ground electronic state. The final step 6 is protonation of the carbanion by HN_3_. (c) Structure of the photocatalyst 4CzIPN and Jablonski diagram schematically showing the sequence (steps A–D) of key photophysical processes for 4CzIPN after 450-nm excitation, as described in the main text. Internal conversion from S_1_ to S_0_, phosphorescence from T_1_, and vibrational relaxation are not included in this simplified scheme.

Organic photocatalysts (OPCs) offer a sustainable and complementary alternative to the archetypal ruthenium- and iridium-based polypyridyl complexes applied in visible-light-mediated synthesis^1−3^ and controlled polymerization.^4−11^ One of many exciting advances in this area is the use of OPCs to drive catalytic derivatizations of C(sp^3^)–H bonds, circumventing the need for prefunctionalized organic substrates.^12−18^ While some OPCs, such as eosin Y,^19^ possess excited states capable of directly abstracting hydrogen atoms from C(sp^3^)–H bonds, a more typical (and modular) approach is to employ a discrete hydrogen-atom transfer (HAT) cocatalyst that may be photooxidized.^14,16^ In this vein, one of our research groups (Cresswell and co-workers) recently discovered that azide ion (N_3_^–^) is an unusually effective HAT catalyst for the challenging photocatalytic α-C–H alkylation of unprotected, primary alkylamines (Figure 1a).^20^ The reported alkylation reactions employed acrylates as coupling partners for the synthesis of γ-amino esters (and their derived γ-lactams),^21^ with subsequent studies extending the chemistry to vinyl phosphonates^22^ and styrenes^20^ as radical acceptors. Cresswell and co-workers proposed the photoredox-HAT cycle depicted in Figure 1b for all of these transformations.

For the initial studies with acrylates, tetrabutylammonium azide (TBAA) was used as an organic-soluble source of the azide ion HAT catalyst, alongside 1,2,3,5-tetrakis(carbazol-9-yl)-4,6-dicyanobenzene (4CzIPN)^23^ as the photocatalyst of choice. Synthesized in 2012 by Adachi and co-workers as a thermally activated delayed fluorescence (TADF) emitter for organic light-emitting diode (OLED) applications,^24−27^ 4CzIPN was first applied to photoredox catalysis by Zhang and co-workers in 2016,^28^ and the use of 4CzIPN and other TADF materials as organophotoredox catalysts has since been reviewed.^29−31^ In a prior study of the photophysics of 4CzIPN, Ishimatsu et al. showed that absorption at wavelengths around 450 nm excites the S_1_ ← S_0_ transition, after which the photoexcited molecules relax *via* competing radiative and nonradiative pathways summarized in Figure 1c.^21,32^ From its S_1_ state, 4CzIPN can fluoresce and return to the ground electronic state, relax nonradiatively by internal conversion (IC) to high vibrational levels of S_0_, or undergo a spin-changing intersystem crossing (ISC) to populate the lowest-lying triplet state (T_1_), with these processes occurring on timescales of a few nanoseconds. In addition, delayed fluorescence is observed on microsecond timescales following reverse intersystem crossing (RISC) from the T_1_ to the S_1_ state.^32^ Both ISC and RISC involve passage through an intermediate triplet excited state (T_2_) not shown in Figure 1c.^33^

The photophysical properties of 4CzIPN depend on solvent polarity. For example, the S_1_ → T_1_ energy gap narrows in polar solvents because of S_1_-state stabilization, enhancing the rate of RISC. However, this greater rate of RISC in polar solvents does not increase the quantum yield for delayed fluorescence but instead promotes S_1_ → S_0_ IC following RISC.^32^ In their aforementioned studies of the α-C–H alkylation of primary amines with acrylate radical acceptors,^21^ Cresswell and co-workers used acetonitrile (MeCN) as a solvent, which should encourage a high RISC rate in the photoexcited 4CzIPN.

In recent studies, we applied two variants of transient absorption spectroscopy, denoted here as transient electronic absorption spectroscopy (TEAS) and transient vibrational absorption spectroscopy (TVAS), to study the mechanisms of photoredox and other reaction cycles over femtosecond to microsecond timescales.^4,6,10,34−39^ Here, we use these methods to examine the multistep mechanism proposed by Cresswell and co-workers^21^ for the photoredox-HAT-catalyzed α-C–H alkylation of cyclohexylamine (CHA) with methyl acrylate (Figure 1a).^40^ From the TEAS and TVAS measurements, we identify reactive intermediates involved in steps 1–3 (Figure 1b) and determine their reaction kinetics. Our assignment of bands observed in the transient spectra to different intermediate species is supported by electronic structure calculations using density functional theory (DFT) methods. These direct spectroscopic observations of the sequential formation and loss of various reactive intermediates reveal some necessary refinements to the proposed reaction mechanism, and they may have implications for the use of azide ion as a HAT catalyst in other transformations.^41−45^

## 2 Methodology

The TEAS data were collected using an ultrafast laser system at the University of Bristol, whereas TVAS data over extended time ranges from sub-picosecond to microsecond were obtained using the LIFEtime facility at the STFC Rutherford-Appleton Laboratory (RAL).^35,46^ The experimental methods used for TEAS and TVAS have been reported previously,^4,5,10,34,36,37,46−53^ and are described in Section S1 in the Supporting Information, together with the methods used for steady-state spectroscopic characterization of samples.

The following experiments were conducted to isolate the mechanistic details of each reaction step. First, a solution of 4CzIPN (synthesized in the University of Bath laboratory)^21,23^ in MeCN was photoexcited at 425 nm or 430 nm and the excited-state dynamics were probed by TVAS and TEAS, respectively. Then, the photochemistry of solutions of 4CzIPN and TBAA in MeCN was studied with TEAS and TVAS methodologies. Finally, photoinduced reactions of mixed solutions of 4CzIPN, TBAA, and cyclohexylamine (CHA; Reagent Plus > 99%) in MeCN were investigated with TVAS. All TVAS experiments were performed in MeCN-d_3_ to minimize interference from strong solvent IR absorption bands. In the experiments reported here, the concentration of 4CzIPN was typically 2.5 mM. Concentrations of TBAA and CHA varied in the ranges 8–40 and 250–920 mM, respectively, so that pseudo-first-order kinetic studies could extract bimolecular rate coefficients for the electron transfer (ET) and HAT reaction steps. The concentration ratio of 4CzIPN:TBAA:CHA in the synthetic studies was 1:10:100, and a similar concentration ratio was used here to emulate the synthetic conditions. Analysis of transient absorption spectroscopy data used the KOALA software package for spectral decomposition (as described in Section S4 in the Supporting Information).^54^

The interpretation of experimental transient absorption spectra made use of predictions from DFT calculations^36,37^ to support the assignment of spectral bands in TEAS or TVAS data. All DFT calculations used the Gaussian 09W software package.^55^Section S2 in the Supporting Information summarizes the methods used. Calculations of HAT reaction pathways used Gaussian 16 (Revision C.01)^56^ as described in Sections S2 and S5 in the Supporting Information.

## 3 Results and Discussion

### 3.1 Steady-State Spectroscopy

**Figure 2:**
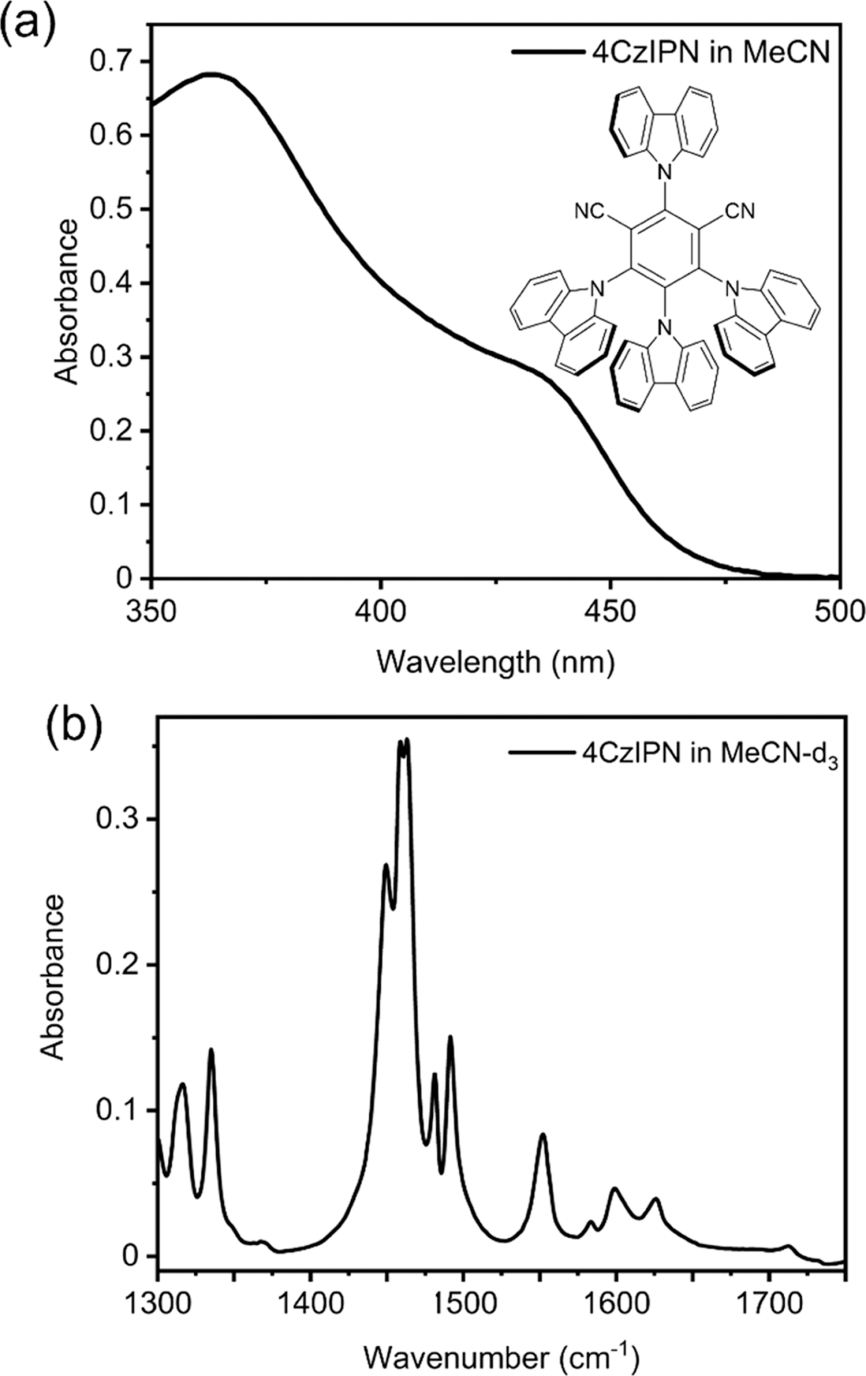
Steady-state spectra of solutions of 4CzIPN in acetonitrile. (a) UV–vis spectrum of a 2.5-mM 4CzIPN solution in MeCN, with a 300-μm sample pathlength. (b) IR spectrum of a 10-mM 4CzIPN solution in MeCN-d_3_, with a 200-μm pathlength.

Figure 2 shows steady-state UV–visible and FTIR spectra of solutions of 4CzIPN in MeCN. Pump wavelengths of 425 nm for TVAS experiments and 430 nm for TEAS experiments were chosen to excite 4CzIPN for consistency with the synthetic chemistry methods used by Cresswell and co-workers.^21^ The long-wavelength shoulder in the 4CzIPN absorption band seen in Figure 2a is assigned to the S_1_ ← S_0_ transition,^32^ with our TD-DFT calculations indicating an oscillator strength *f* = 0.1336. At our chosen excitation wavelengths, some initial vibrational excitation is expected for the 4CzIPN population in the S_1_ state. No other reacting species in the mixed solutions absorbed the 425–430 nm excitation light, as shown by steady-state spectra in Section S1, Figure S1 in the Supporting Information.

The IR absorption spectrum of the 4CzIPN in MeCN-d_3_ solution, as seen in Figure 2b for the 1300–1750 cm^–1^ region, contains bands attributed to aromatic C=C stretching and C–N stretching modes in the heterocyclic rings. These IR bands are used to assign ground-state bleach (GSB) features observed in TVA spectra for solutions containing 4CzIPN. IR spectra of all reactants can be found in Section S1 Figure S2 in the Supporting Information.

### 3.2 Transient Absorption Spectroscopy of 4CzIPN in Acetonitrile Solution

**Figure 3:**
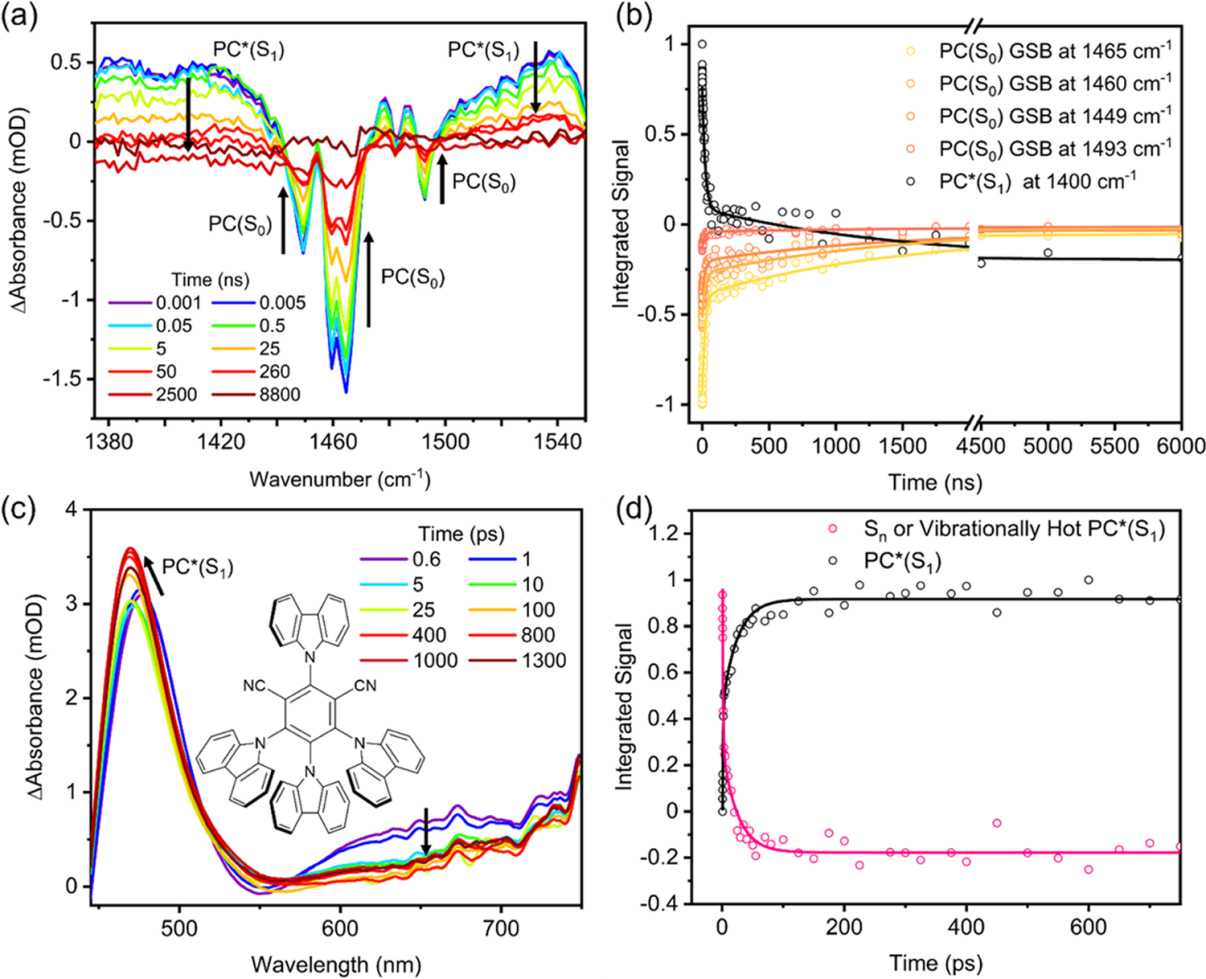
Transient absorption spectra and derived kinetic traces. (a) TVA spectra of 2.5-mM 4CzIPN in MeCN-d_3_ excited at 425 nm; (b) kinetic traces obtained from analysis of band intensities in the TVA spectrum in (a); (c) TEA spectra of 2.5-mM 4CzIPN in MeCN excited at 430 nm, with the ESA feature at wavelengths above 600 nm discussed in the main text; and (d) kinetic traces for the main bands in the TEA spectrum shown in (c). The negative offset to the intensity of the short-lived ESA feature at later times is a consequence of the choice of fitting functions used to decompose the TEA spectra (see Figure S14 in the SI).

TVA and TEA spectra were collected for 2.5-mM solutions of 4CzIPN in MeCN (TEAS) or MeCN-d_3_ (TVAS) following 425-nm (430-nm for TEAS) photoexcitation, and examples are shown in Figure 3. The TVA spectra were obtained over the wavenumber interval 1350–2200 cm^–1^. In the 1370–1550 cm^–1^ region shown in Figure 3a, positive features in the wavenumber intervals 1370–1430 and 1500–1550 cm^–1^ correspond to vibrational bands of the initially excited 4CzIPN photocatalyst, PC*(S_1_). Negative-going features at 1449, 1460, 1465, 1482, and 1493 cm^–1^ instead correspond to ground-state bleaches (GSBs) representing the depletion of the 4CzIPN photocatalyst PC(S_0_) ground-state population. PC*(S_1_) also shows broad excited-state absorption (ESA) bands extending from ∼1800 to 2050 cm^–1^ (see Figure S15 in the Supporting Information) where there are no corresponding ground-state absorption features. The kinetics of PC*(S_1_) decay and PC(S_0_) recovery were extracted from these data in KOALA by integrating the transient absorption intensities in the wavenumber region 1370–1430 cm^–1^, or by fitting Gaussian functions to resolved GSBs, respectively. To fit the broad ESA bands for data in the range 1800–2050 cm^–1^, a basis function corresponding to the early time spectrum was used because the band shape did not vary over the temporal range of the experimental measurements. Examples of these spectral decompositions can be found in Section S4 in the Supporting Information.

The kinetics of changing band intensities revealed by TVAS measurements were analyzed to obtain exponential time constants that characterize the photophysics of 4CzIPN in MeCN-*d*_3_. After a fast (τ_1_ ≤ 50 ps) initial decay component likely to represent vibrational cooling of the photoexcited PC*(S_1_) molecules, time-dependent band intensities were globally fitted to a biexponential function for the decay of PC*(S_1_) ESA and recovery of PC(S_0_) GSB features. The two time constants obtained for the decay of PC*(S_1_), or for recovery of PC(S_0_), are τ_2_ = 21.5 ± 1.0 ns and τ_3_ = 1500 ± 170 ns. The τ_2_ time constant accounts for a combination of IC from S_1_ to S_0_, prompt decay of S_1_ population by fluorescence, and ISC from the lowest vibrational levels of S_1_ to the manifold of triplet states, whereas τ_3_ is the time constant for delayed fluorescence and IC resulting from RISC. These values are in reasonable agreement with Ishimatsu *et al.* who reported time constants for prompt and delayed fluorescence from 4CzIPN in MeCN of τ_2_ = 18.7 ns and τ_3_ = 1390 ns, with unspecified uncertainties.^32^ The TVA spectral band intensities in the interval 1800–2050 cm^–1^ show similar kinetics (see SI Figure S15).

TEAS data such as those shown in Figure 3c reveal two ESA features. A rapidly decaying peak in the wavelength range 600–750 nm is likely to be absorption from excited vibrational levels of the S_1_ state of 4CzIPN, or from a higher-lying singlet state (S_n_) also populated by absorption from S_0_ at the chosen excitation wavelength of 430 nm. A stronger and longer-lived feature is initially centered at 477 nm and shifts to 470 nm over time, which is indicative of vibrational relaxation. The 470-nm ESA band is attributed to absorption from vibrationally relaxed PC*(S_1_) species. It is unlikely to correspond to absorption from PC*(T_1_) because ISC predominantly occurs outside the temporal window accessible in the TEAS measurements (see above). The kinetics extracted from the analysis of these transient spectra are shown in Figure 3d, and a global biexponential fit gives time constants of 1.0 ± 0.1 and 25 ± 2 ps. The former may correspond to IC from an S_n_ state to S_1_, whereas the latter is most likely to be a consequence of the vibrational relaxation of internally excited PC*(S_1_) molecules, consistent with the τ_1_ values obtained from TVAS measurements. The fits to the time-dependent band intensities were limited to data obtained for delays up to 750 ps; thereafter, indications of PC*(S_1_) population decay were observed.

### 3.3 Transient Absorption Spectroscopy of 4CzIPN and TBAA Solutions in Acetonitrile

**Figure 4:**
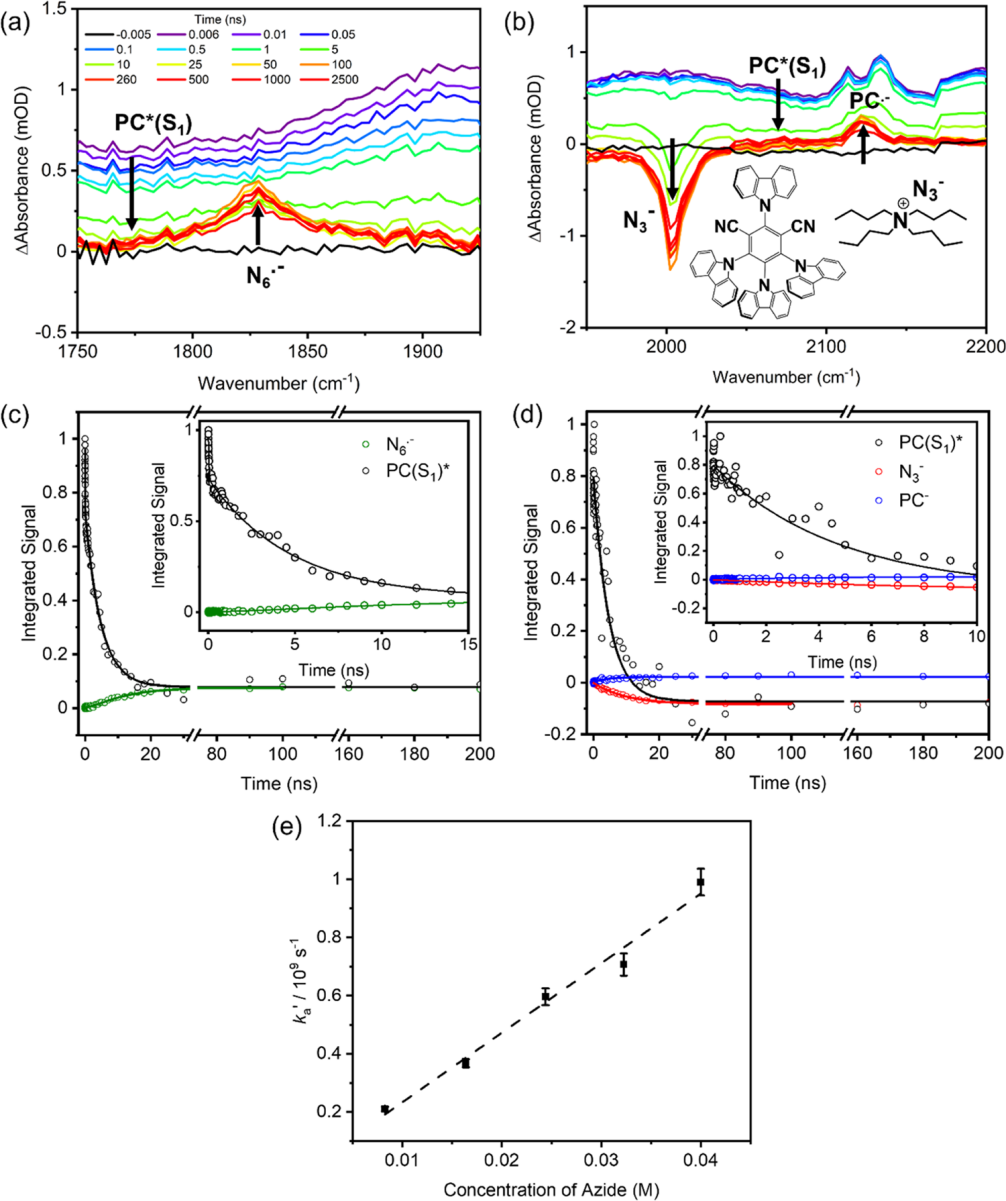
Kinetics of the electron transfer reaction of PC* with N_3_^–^. (a, b) TVA spectra spanning different wavenumber intervals for a solution of 2.5-mM 4CzIPN and 8-mM TBAA in MeCN-d_3_. The inset color scheme identifies spectra obtained at different time delays, and black arrows indicate the directions of change of the band intensities. The black lines are baseline spectral measurements obtained at a negative time delay (with the probe pulse before the excitation pulse). (c, d) The corresponding integrated band intensities and fits to kinetic models described in the main text. (e) Pseudo-first-order kinetic plot for the rate coefficient *k*_a_′ (see main text) quantifying the decay of PC*(S_1_) at various concentrations of TBAA (black squares), and an unweighted linear fit (dashed line).

To study the electron transfer reactions of photoexcited 4CzIPN with azide (N_3_^–^) ions, MeCN-*d*_3_ solutions of 2.5-mM 4CzIPN and varying concentrations of TBAA in the range 8–40 mM were prepared, with concentrations chosen to replicate the synthetic molar ratios used by Cresswell and co-workers.^21^Figure 4 shows the resultant TVA spectra and kinetic traces at 8 mM TBAA concentration, obtained using photoexcitation of the 4CzIPN at 425 nm.

According to the mechanism proposed by Cresswell and co-workers (Figure 1),^21^ the reaction between TBAA and photoexcited PC* forms an azidyl radical and PC^•–^ radical anion. To observe this reaction in the TVAS measurements, we chose the mid-IR region from 1300 to 2200 cm^–1^ because our DFT calculations predicted 4CzIPN anion bands in this region, and the N_3_^•^ radical was previously shown to have a band centered at 1658 cm^–1^.^57^ In the 1375–1550 cm^–1^ interval, the ESA bands attributed to PC*(S_1_) (Section 3.2) are again seen in all TVA spectra at early times, but now they decay to baseline on a few nanosecond timescale (see Figure S16 in the SI), with commensurate growth of a PC^•–^ radical anion absorption band. This observation suggests that the bimolecular electron transfer from N_3_^–^ to PC* (step 2 in Figure 1) involves the PC*(S_1_) state, as opposed to the longer-lived PC*(T_1_) state, under these experimental conditions. Some measurements also reveal a shorter-lived spectral component that decays with a picosecond time constant. This fast-decay component is not thought to be the result of static electron transfer between N_3_^–^ and PC* because a transient IR band discussed below and assigned to the PC^•–^ radical anion product does not show a similarly fast component of growth.

Figure 4a,b instead shows the evolution of transient vibrational absorption spectra in the 1750–2200 cm^–1^ wavenumber interval. With the inclusion of TBAA in the reaction mixture, three additional peaks appear in the TVA spectra as the broad PC* absorption decays: two positive peaks are located at 1829 and 2125 cm^–1^, and a GSB is centered at 2005 cm^–1^. With the support of DFT calculations, the 2125 cm^–1^ band is assigned to absorption by the PC^•–^ radical anion product of the ET reaction, whereas the associated N_3_^–^ depletion accounts for the GSB feature at 2005 cm^–1^. However, the assignment of 1829 cm^–1^ proved to be more involved. The proposed mechanism for the catalytic cycle shown in Figure 1 suggests that the azidyl radical should be observable in the TVAS data after ET from N_3_^–^ to PC*(S_1_). However, the known IR absorption band for N_3_^•^ at 1658 cm^–1^ is not seen (see Figure S17 in the SI for an example set of spectra).^58^ The wavenumber of this expected N_3_^•^ band is reasonably well reproduced by DFT calculations reported in Section S3, Figure S8 in the Supporting Information. The observed 1829 cm^–1^ band cannot be plausibly attributed to an overtone or combination band of N_3_^•^; instead, it is assigned to the N_6_^•–^ radical anion, which is known to form by the association of N_3_^•^ with N_3_^– ^^44,58−63^
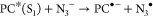
1
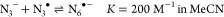
2Pulse-radiolysis studies of N_3_^•^ HAT reactions in aqueous solution showed the equilibrium to favor N_3_^•^ (K = 0.33 M^–1^),^62^ but in MeCN, the association to N_6_^•–^ dominates (*K* = 200 M^–1^).^58,60^ Our assignment of the 1829 cm^–1^ band to N_6_^•–^ for our experiments in MeCN is supported by previous computational and spectroscopic studies, and by our DFT calculations.^58,60^ Workentin et al. computed the lowest-energy structure of N_6_^•–^ to be a square, cyclic arrangement of the N_3_^•^ and N_3_^–^moieties, as shown in Section S3 in the Supporting Information.^58^ Our observations confirm that N_3_^•^ radicals formed by reaction 1 rapidly associate with the excess N_3_^–^ ions from TBAA dissociation in solution, and this interpretation raises the possibility that the resulting N_6_^•–^ radical anions are responsible for the activation of amines by HAT in step 3 of the photoredox cycle (Figure 1).

The data shown in Figure 4 are for a solution of 2.5-mM 4CzIPN and 8-mM TBAA in MeCN. Corresponding data for other concentrations of TBAA are included in Section S4 in the Supporting Information. Figure 4c shows plots of the extracted time-dependent band intensities from the analysis of the spectra in Figure 4a. Integrated band intensities were extracted by fitting to a basis function representing PC*(S_1_) absorption (taken from an early time transient spectrum), and a Gaussian function to model the feature at 1829 cm^–1^ revealing N_6_^•–^ growth.

A biexponential fit to the PC*(S_1_) ESA decay yields time constants with values of 35 ± 4 ps and 4.8 ± 0.2 ns. The smaller time constant is assigned to vibrational relaxation in the PC*(S_1_) state (see above), and perhaps a component of prompt ISC from vibrationally excited S_1_ molecules.^37^ The value of the second time constant is determined by a combination of ISC and diffusive ET reaction between PC*(S_1_) and N_3_^–^; hence, it depends on the concentration of TBAA. The kinetics of growth of the N_6_^•–^ absorption band are in accordance with expectations for an intermediate in a sequential reaction scheme, as expressed in eqs 3–5. Our notation for rate coefficients and time constants uses subscript letters *a* and *b* for bimolecular reactions in the presence of N_3_^–^ to distinguish them from the time constants τ_1_–τ_3_ for photochemical processes in solutions of 4CzIPN without added TBAA (see Section 3.2).
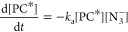
3
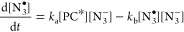
4
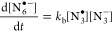
5Because the concentration of N_3_^–^ is in excess over PC* and N_3_^•^, the decay of PC* and growth of N_6_^•–^ will follow pseudo-first-order kinetics. We denote the corresponding pseudo-first-order rate coefficients with a prime, for example, *k*_a_′ = *k*_a_[N_3_^–^]. In accordance with this model, the rate of growth of the N_6_^•–^ band will depend on both the rate of decay of PC*(S_1_) population and the concentration of N_3_^–^ (or, equivalently, TBAA).

Figure 4d shows the time-dependent band intensities obtained from the analysis of spectra in Figure 4b, together with kinetic fits. Integrated band intensities were obtained from fits incorporating PC*(S_1_) absorption (using an early time spectrum as a basis function), and Gaussian functions for the features at 2005 and 2125 cm^–1^ representing N_3_^–^ depletion and PC^•–^ growth, respectively. The fits for N_6_^•–^ growth extend to a maximum time delay of 100 ns because at later time, the N_6_^•–^ absorption begins to decay. To make the fitting robust for the kinetics of growth of N_6_^•–^ in an 8-mM TBAA solution, the first time constant (τ_a_ = 1/*k*_a_′) was fixed to the value of τ_a_ = 4.8 ns derived from analysis of the PC*(S_1_) decay, as discussed above, while the second time constant was allowed to vary, giving τ_b_ = 1/*k*_b_′ = 8.4 ± 0.9 ns. Global, monoexponential fits of the decay of PC*(S_1_) and the growth of PC^•–^ absorption bands in the range 1930–2215 cm^–1^ were consistent with this τ_a_ = 4.8 ns value. The kinetics of loss of N_3_^–^ via reaction 2, monitored by the depth of the 2005 cm^–1^ depletion feature evident in Figure 4b, are more difficult to interpret because the TBAA is in excess. However, to confirm that the kinetics are dependent on 4CzIPN photoexcitation and N_6_^•–^ formation, the first 100 ns of N_3_^–^ depletion can be satisfactorily accounted for by a biexponential fit with fixed time constants τ_a_ = 4.8 ns and τ_b_ = 8.4 ns.

For different concentrations of the TBAA, time constants for the loss of PC*(S_1_) obtained from analysis of TVAS data in the range 1735–1925 cm^–1^ were converted into pseudo-first-order rate coefficients, *k*_*a*_′. Figure 4e shows a pseudo-first-order plot for the kinetics of PC(S_1_)* loss by ET reaction with N_3_^–^, together with a linear fit to extract a value for the second-order rate coefficient, *k*_a_ = (2.4 ± 0.2) × 10^10^ M^–1^ s^–1^. This value is close to the estimated diffusion-limited rate coefficient *k*_diff_ = 1.9 × 10^10^ M^–1^ s^–1^ for an MeCN solution at 25 °C.^64^ A pseudo-first-order kinetic analysis was not performed for the growth of N_6_^•–^ because, at higher concentrations of TBAA, the *k*_a_′ and *k*_b_′ values were too similar to separate reliably.

**Figure 5:**
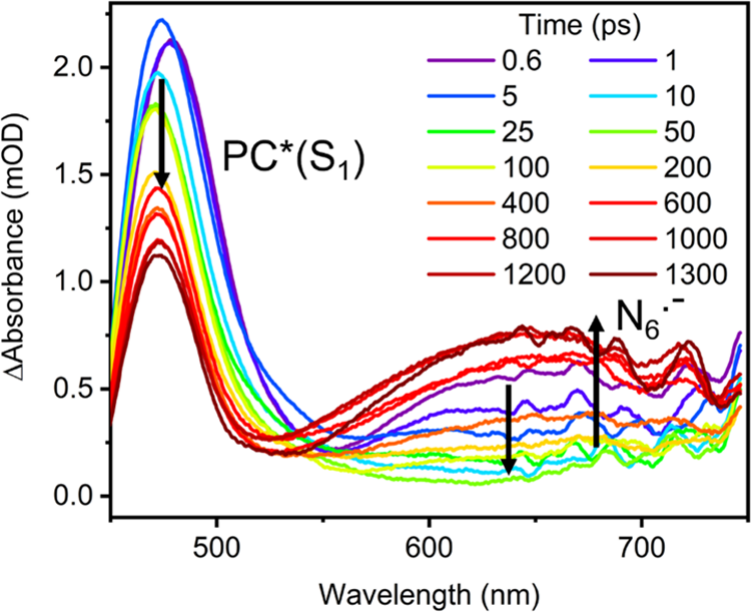
TEA spectra of an acetonitrile solution of 1.1-mM 4CzIPN and 170-mM TBAA photoexcited at 430 nm. The inset color scheme identifies spectra obtained at different time delays, and black arrows indicate the directions of change of the band intensities. The growth of a broad absorption band at wavelengths from 550 to 750 nm at time delays greater than 200 ps is attributed to the production of N_6_^•–^ radical anions via reaction 1. The initial decay in ESA for wavelengths above 580 nm indicated by a black downward arrow is also observed for 4CzIPN solutions in MeCN in the absence of TBAA (Figure 3c) and is discussed in Section 3.2.

To observe the reaction between PC* and TBAA by TEAS in the limited temporal window available to our experiments, a higher concentration of TBAA (170 mM) was necessary. The TEA spectra for the reaction of PC* with TBAA resemble those presented in Section 3.1, although with faster decay of the band assigned to PC*(S_1_) ESA and growth of an additional peak centered at about 650 nm, as illustrated in Figure 5. The wavelength of this new band is consistent with reactions 1 and 2 forming N_6_^•–^, which has a known electronic absorption band in this spectral region.^58−60^

### 3.4 Transient Absorption Spectroscopy of Solutions of 4CzIPN, TBAA, and CHA in Acetonitrile

**Figure 6:**
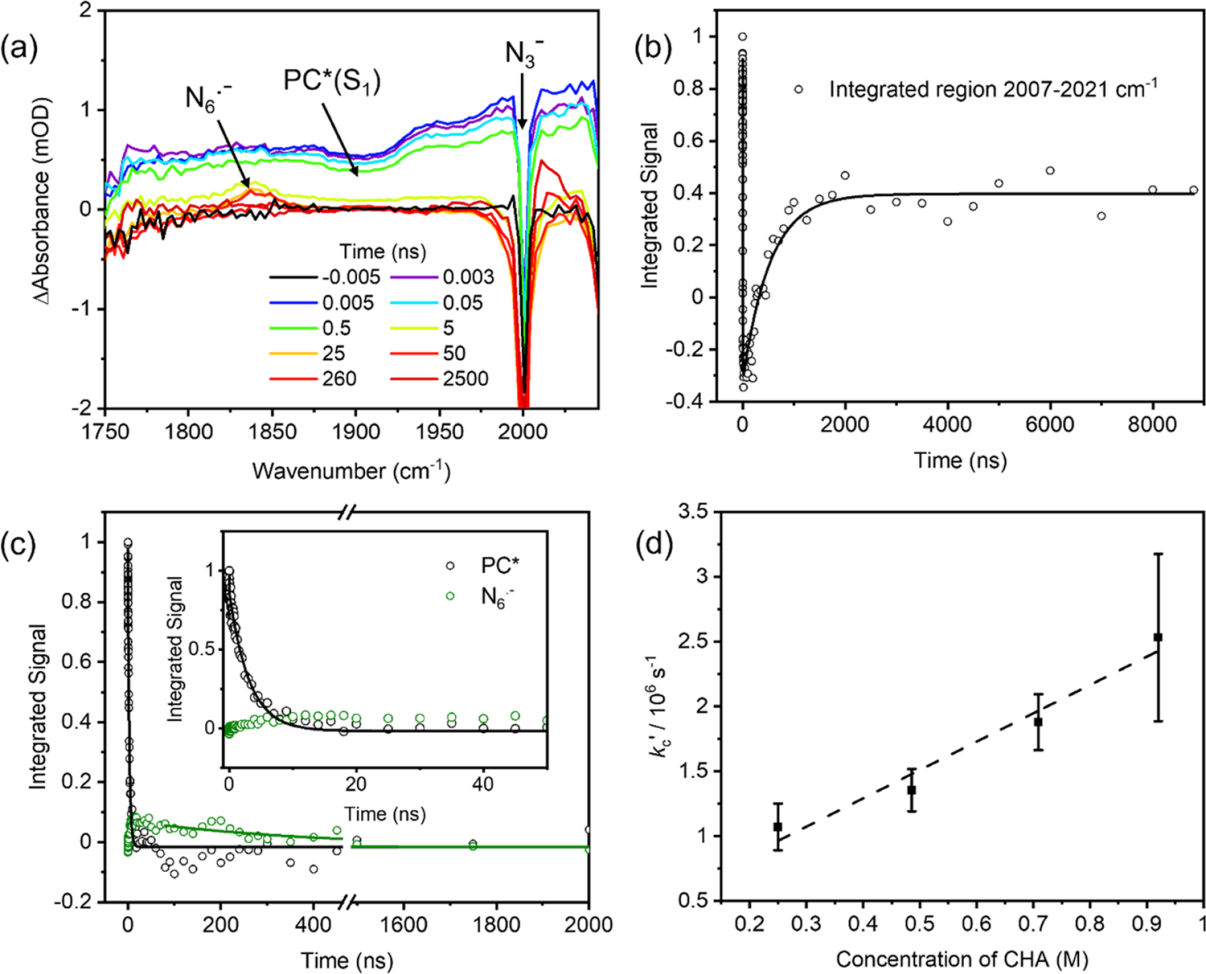
Kinetics of reaction of N_6_^•–^ with CHA. (a) TVA spectra for a solution of 1.6 mM 4CzIPN, 17 mM TBAA, and 920 mM CHA in MeCN-d_3_. The black line is a baseline spectrum obtained at a negative time delay. (b, c) Integrated intensities for various observed bands (open circles) and fits to kinetic models described in the main text (solid lines). Negative-going signals in (c) are a consequence of overlap with a strong azide ion band. (d) Pseudo-first-order kinetic plot for the decay of the N_6_^•–^ absorption band intensity, and a linear fit (dashed line) with no error weighting.

The addition of CHA to the reaction mixture allows the H-atom transfer reaction identified as step 3 in Figure 1 to be studied using TVAS. Solutions of 1.6–1.8 mM 4CzIPN, 17–19 mM TBAA, and 250–920 mM CHA were prepared in MeCN-*d*_3_. The solutions were photoexcited at 425 nm and probed by TVAS over extended timescales. Figure 6 shows an example data set and the associated kinetic analysis.

At time delays up to a few hundred nanoseconds, the transient absorption spectra (Figure 6a) resemble those described in Section 3.3. However, the peak at 1829 cm^–1^ identified as arising from N_6_^•–^ and growing on a ns timescale now decays more rapidly at longer times, plausibly because of reaction with CHA (with bimolecular rate coefficient *k*_c_)
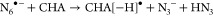
6Here, CHA[-H]^•^ denotes the radical formed by H-atom abstraction from CHA. This reaction regenerates N_3_^–^ ions. Growth of an additional peak centered near 2014 cm^–1^ is observed to the higher-wavenumber side of the N_3_^–^ GSB feature at 2005 cm^–1^. The observed kinetics suggest a possible assignment is to the CHA[-H]^•^ radical, but our DFT calculations (Figure S7 in the Supporting Information) do not identify any CHA[-H]^•^ absorption bands in this region. Integrated band intensities were derived from fits to an early time spectrum, used as a basis function to represent PC*(S_1_) absorption, and a Gaussian function to describe the feature at 1829 cm^–1^ attributed to N_6_^•–^. Integration from 2007 to 2021 cm^–1^ was used to extract time-dependent transient absorption intensities in this wavenumber interval.

The kinetics of N_6_^•–^ decay were approximately monoexponential for time delays after the growth of this radical anion was complete, as exemplified in Figure 6c. The N_6_^•–^ growth and decay occur on very different timescales, so their kinetics can be separated in this way, with a fit resulting in a time constant for N_6_^•–^ decay of τ_c_ = 395 ± 100 ns for a 920 mM CHA solution. A CHA concentration-dependent study was undertaken, and the pseudo-first-order kinetic analysis shown in Figure 6d gives *k*_c_ = (2.2 ± 0.3) × 10^6^ M^–1^ s^–1^. This rate coefficient for reaction 6 is 4 orders of magnitude smaller than the diffusion limit, suggesting activation control because of an energy barrier to H-atom transfer.

Fitting the time-dependent changes in absorbance in the 2007–2021 cm^–1^ interval (Figure 6b) required a triexponential function, with the first two time constants constrained to those previously determined for PC*(S_1_) decay (the ESA from which extends across this integration region). A best-fit value τ_d_ = 532 ± 46 ns was derived for the third component when the concentration of CHA was 920 mM. Because this band grows more slowly than the loss of N_6_^•–^ and cannot be assigned to CHA[-H]^•^, it is suggested to arise from further reaction of CHA[-H]^•^ in our solutions.

To explore further the possible reactivity of N_6_^•–^, quantum chemical calculations for this radical anion were performed using methods described in Section S2 in the Supporting Information. The resulting structures and energies from all calculations are reported in Section S5 in the SI. N_6_^•–^ was calculated to be favored relative to N_3_^•^ + N_3_^–^ (by a Gibbs energy of 38.9 kJ mol^–1^ in acetonitrile), which is in agreement with previous experimental and computational studies of this equilibrium.^58^ Four structures for the N_6_^•–^ species were evaluated, with the lowest energy being the planar symmetric structure in which two N_3_ fragments are connected by two long bonds to form a rectangle, as reported in a previous computational study.^58^

The α-NH_2_ HAT barrier for the reaction of N_3_^•^ with cyclohexylamine in acetonitrile was calculated previously to be 19.6 kJ mol^–1^.^21^ In our current work, despite extensive efforts, TSs for α-NH_2_ HAT with N_6_^•–^ and cyclohexylamine in acetonitrile could not be found at the same level of theory as the previous study. Instead, these TSs began optimizing toward α -NH_2_ HAT TSs in which one N_3_ fragment moved away from the reaction site. We therefore conclude that N_3_^•^ is the key reactive radical in the HAT reactions, but that complexation with N_3_^–^ to make N_6_^•–^ regulates the concentration of free N_3_^•^ in MeCN solution.

In addition to reactions in acetonitrile of the type considered here, Cresswell and co-workers have also successfully applied the α-NH_2_ HAT chemistry for reactions in THF solution. The success of this chemistry with THF as the solvent raises the question of why the α-O HAT reaction of N_3_^•^ with the THF does not compete with the desired solute amine reactions. Drawing on the observations from transient absorption spectroscopy reported here, one posited explanation was a greater role for the reaction of the N_6_^•–^ complex in THF. However, calculations for the α-O HAT reaction of N_3_^•^ with THF instead point to a more straightforward explanation. The computed barrier to α-O HAT reaction of N_3_^•^ with THF in MeCN solution is 43.8 kJ mol^–1^, and with implicit THF solvation, this barrier is 44.3 kJ mol^–1^. The higher computed HAT barrier for THF therefore accounts for the reaction of N_3_^•^ with cyclohexylamine being preferred over reaction with the THF solvent, without the need to invoke reactivity of N_6_^•–^.

## 4 Conclusions

Transient absorption spectroscopy on sub-picosecond to microsecond timescales has been used to observe directly the intermediates involved in three consecutive steps in a photoredox-catalyzed cycle recently developed for α-C–H alkylation of unprotected primary alkylamines with acrylate Michael acceptors.^21^ The excited-state dynamics of the organic photoredox catalyst 4CzIPN were studied using complementary TEAS and TVAS methods, and its relaxation pathways were observed, with the kinetics of RISC matching those reported previously from time-resolved photoluminescence measurements.^32^ The kinetics of electron transfer from azide anions (present as dissolved TBAA) to photoexcited 4CzIPN were determined directly by observing the rate of loss of excited-state absorption by the 4CzIPN. For the TBAA concentrations used in TVAS studies, which are comparable to those employed in the photoredox reactions of Cresswell and co-workers,^20−22^ the electron transfer reaction involves the S_1_ excited state of 4CzIPN, and not the longer-lived T_1_ state. This bimolecular ET reaction was found to be diffusion-limited, consistent with a large thermodynamic driving force for electron transfer from N_3_^–^ to the orbital vacancy created by electronic excitation of 4CzIPN. Interestingly, the azidyl (N_3_^•^) radicals formed by the electron transfer did not remain as reactive species in significant concentrations, but instead rapidly associated with excess N_3_^–^ to form cyclic N_6_^•–^ ions,^58−60^ which are analogues of the X_2_^•–^ radical anions formed by halogen atoms (X^•^) in solutions containing halide ions (X^–^). The N_6_^•–^ was identified by its distinctive IR band at 1829 cm^–1^ and a broad electronic absorption band at wavelengths around 650 nm.

The addition of cyclohexylamine to the reaction mixture induced decay of the N_6_^•–^ concentration because of a bimolecular H-atom transfer reaction determined to have a rate coefficient 4 orders of magnitude smaller than the diffusion limit. However, quantum chemistry calculations of HAT reaction barriers point to N_3_^•^ being the reactive species, while N_6_^•–^ constitutes a reservoir that regulates the concentration of free N_3_^•^ radicals. There were no definitive signs of the formation of CHA[-H]^•^ radicals in the transient absorption spectra. The direct observations of almost all of the intermediates involved in three critical steps of a photoredox-catalyzed reaction cycle provide both kinetic data and confirmation, with some important refinements, of the previously proposed reaction mechanism.^21^

## Data Availability

Data are available at the University of Bristol data repository, data.bris, at https://doi.org/10.5523/bris.10lppiug4bgdk2nljkbto8qm73.

## Supporting Information Available

Experimental methodology; steady-state absorption spectra; computational methodology; comparison of measured and computed spectra; decomposition of transient absorption spectra; kinetic data; and computational data (PDF)
