# Supplementary material for: Photoredox-HAT Catalysis for Primary Amine α-C–H Alkylation: Mechanistic Insight with Transient Absorption Spectroscopy
Source: ACS Catal. 2023 May 30;13(12):8004–13. doi: 10.1021/acscatal.3c01474 (PMC10278065; doi:10.1021/acscatal.3c01474)
Supplement: Supplementary file 1 — cs3c01474_si_001.pdf [file cs3c01474_si_001.pdf]

Photoredox-HAT Catalysis for Primary Amine α-C–H Alkylation: Mechanistic Insight with Transient Absorption Spectroscopy

Mahima Sneha[a,b] *#, Georgia L. Thornton[a#], Luke Lewis-Borrell[a], Alison S. H. Ryder[c], Samuel G. Espley[d], Ian P. Clark[e], Alexander J. Cresswell[d], Matthew N. Grayson[d], Andrew J. Orr-Ewing[a] *

[a] School of Chemistry, University of Bristol, Cantock's Close, Bristol, BS8 1TS, UK.

[b] Department of Chemistry, Dartmouth College, Hanover, NH 03755, USA.

[c] Centre for Sustainable Chemical Technologies, University of Bath 1 South, Claverton Down, Bath, BA2 7AY, UK.

[d] Department of Chemistry, University of Bath 1 South, Claverton Down, Bath, BA2 7AY, UK.

[e] Central Laser Facility, Research Complex at Harwell, Science and Technology Facilities Council, Rutherford Appleton Laboratory, Harwell Oxford, Didcot, OX11 0QX, UK.

# These authors contributed equally.

*Authors for Correspondence: a.orr-ewing@bristol.ac.uk, mahima.sneha@dartmouth.edu

Index

	Page
**Section S1 Experimental Methodology and Steady-State Absorption Spectra**	**S3**
**Section S2 Computational Methodology**	**S8**
**Section S3 Comparison of Measured and Computed Spectra**	**S10**
**Section S4 Decomposition of Transient Absorption Spectra and Extracted Kinetic Data**	**S14**
**Section S5 Computational Data**	**S22**

Section S1 Experimental Methodology and Steady-State Absorption Spectra

S1.1 Transient absorption spectroscopy

Transient absorption spectra were measured for samples circulated by a peristaltic pump in a continuous-flow system through a Harrick cell fitted with two CaF_2 windows separated by 200 or 300 µm PTFE spacers. The cell position was constantly rastered in the plane perpendicular to the laser beam propagation direction to prevent photodamage to 4CzIPN and photodegraded sample deposition on the cell windows. All the experimental measurements were conducted at an ambient laboratory temperature of 20 ^{o}C, both at the University of Bristol and RAL laboratories.

Because the TVAS measurements sampled extended time delays, special care was taken to remove any oxygen contamination which could quench the population of triplet states or lead to unwanted side reactions. Solutions were prepared in an amber Duran bottle attached to a sealed flow system. Solutions used degassed solvents and were sparged with nitrogen gas before use. The headspace of the Duran bottle and the flow-system PTFE tubing were flushed with nitrogen prior to sealing. Nevertheless, it was difficult to exclude oxygen leaks completely from the flow system.

S1.1.1 TVAS data collection

All TVAS data reported here were obtained using the LIFEtime facility at RAL.[1-6] In the LIFEtime laser system, the 1030-nm output of a laser oscillator (80 MHz) seeded two Yb:KGW amplifiers (Light Conversion, Pharos, 15 W, 100 kHz, 260 fs output and Light Conversion, Pharos SP, 6W, 100 KHz, 180 fs). The Pharos amplifier pumped an optical parametric amplifier (OPA; Light Conversion, Orpheus HP) to generate the UV-Vis pump excitation wavelength, and the Pharos SP amplifier pumped two OPAs (Light Conversion, Orpheus ONE) from which independently tuneable mid infra-red (IR) probe beams were obtained by difference frequency generation. The two probe beams were focused by a f = 7.5 cm gold parabolic mirror to achieve identical spot sizes. The repetition rate of the UV pump pulses was reduced to 1 kHz by pulse picking to implement multiple-probe-pulse measurements following each excitation pulse. The timing between the UV-Vis pump pulses and the multiple IR probe pulses was controlled using a combination of a 0 – 16 ns optical delay stage and electronic pulse selection, giving efficient

measurement of time delays from <1 ps – 100 μs. The pulse energy for the 425 nm UV-Vis pump was below 300 nJ/pulse to avoid any multiphoton excitations, and both IR probe beam energies were 50 nJ/pulse.

For TVAS measurements, the UV-Vis pump and two IR probe beams were spatially overlapped at the sample and the transmitted IR beams were separately dispersed by gratings onto a pair of 128-element MCT array detectors (InfraRed Associates, Inc.) to generate a TVA spectrum. The probe IR region was selected in the range 1350 – 2210 cm^{-1}, with the centre wavenumbers of the two probe pulses detuned to cover separate spectral windows and each pulse spanning approximately 200 cm^{-1}. Reference spectra of polystyrene and 1,4-dioxane were used to calibrate the pixel-to-wavenumber conversion of the TVAS data after acquisition.

S1.1.2 TEAS data collection

TEAS measurements used a combination of an ultrafast laser oscillator (Coherent Vitara-S) and amplifier (Legend Elite HE+) to output 800-nm laser pulses of duration 55 fs with a repetition rate of 1 kHz and a total power of 4.5 – 5.0 W. A beam splitter separated 2% of the output power to generate a white light continuum (WLC) probe beam for TEAS experiments. The remaining 98% was divided by a 50:50 beam splitter to pump two OPAs, one of which was used to generate 430-nm pump pulses for 4CzIPN excitation. The OPA was tuned to 430 nm instead of 425 nm to circumvent poor conversion efficiency at 425 nm.

A half-wave plate and a linear polarizer were used to attenuate the energy of the 430-nm light to approximately 130 nJ per pulse. The polarizer was set at the magic angle (54.7°) relative to the polarization of the probe pulses to eliminate anisotropy effects. A retroreflector mounted on a moveable stage controlled the time delays between the 430-nm pulses and the WLC probe pulses up to a maximum of 1.3 ns. A 500-Hz mechanical chopper blocked every second pulse of the 430-nm beam for comparison of pump-on and pump-off measurements to generate TEA spectra.

The WLC and 430-nm beams were focused into the sample using a *f* = 75-mm off-axis concave aluminium mirror and a *f* = 200-mm CaF_2 lens, respectively, to maintain a pump beam size twice

the size of the WLC probe. After the sample, the 430-nm beam was blocked and the WLC was re-collimated before passing through a 2-mm cuvette containing a copper sulphate solution (≥ 99.9%, VWR Chemicals) to remove any residual 800-nm light. The transmitted WLC was then focused into an Andor spectrometer (Shamrock 163) fitted with a 1024-element photodiode array (Entwicklungsbüro Stresing) to obtain a TEA spectrum. Holmium oxide glass was used to calibrate the pixel-to-wavelength conversion for the transient spectra.

S1.1.3 TEAS and TVAS data analysis

All TEAS and TVAS data were analysed using the KOALA software package[7] to extract kinetic traces that were fitted in Origin software to obtain time constants for the various mechanistic steps. Additional information about the fitting methods and extracted data can be found in Section S3 of the Supporting Information.

S1.2 Steady-state spectroscopy.

The steady-state UV-Vis and FTIR absorption spectra of 4CzIPN, Cyclohexylamine, and Tetrabutylammonium azide were obtained with a Thermo Scientific Genesys 10S spectrophotometer and a PerkinElmer Spectrum-Two spectrometer. The measurements were made using a stainless-steel Harrick cell with CaF_2 windows, and a sample path length of 200 – 300 μm was used for both UV and IR spectroscopy. For UV-Visible spectroscopy, a 4CzIPN solution was prepared in acetonitrile to a concentration such that it exhibited an absorbance of $0.05 < A(\lambda) < 0.5$ OD at the excitation wavelength λ = 420-430 nm. To ensure that TBAA and CHA did not absorb at this wavelength, UV-Vis spectra were obtained of their solutions at concentrations of 25 mM and 250 mM, respectively, to replicate the molar ratios used in the transient absorption and synthetic chemistry experiments. Example UV-Vis spectra are shown in Figure S1. FTIR spectra were measured in acetonitrile-d_3 to eliminate solvent absorption bands in the region between 1000-4000 cm^{-1}, and Figure S2 shows IR bands of interest to the current study.

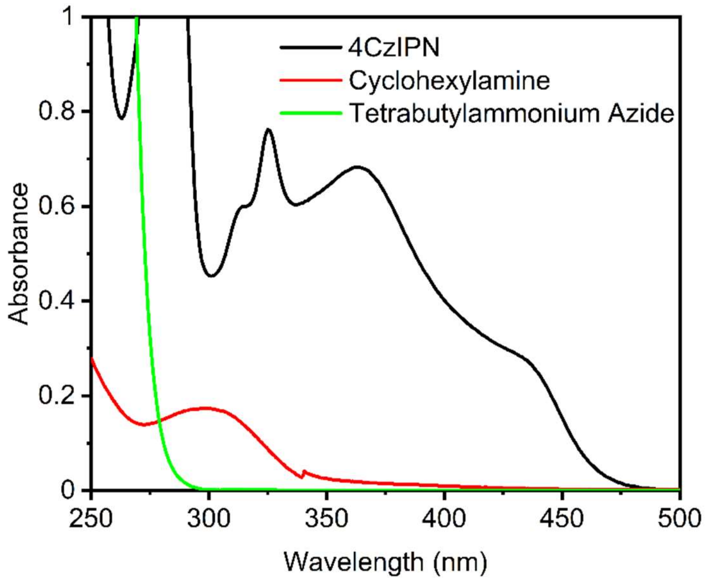

Fig. S1 *Overlaid UV-Vis spectra of solutions of 2.5 mM 4CzIPN, 250 mM CHA and 25 mM TBAA, each in acetonitrile (MeCN). Spectra were obtained in a Harrick cell with a 300-µm sample pathlength. Only 4CzIPN has significant absorption at the 425-430-nm excitation wavelengths used in the current studies.*

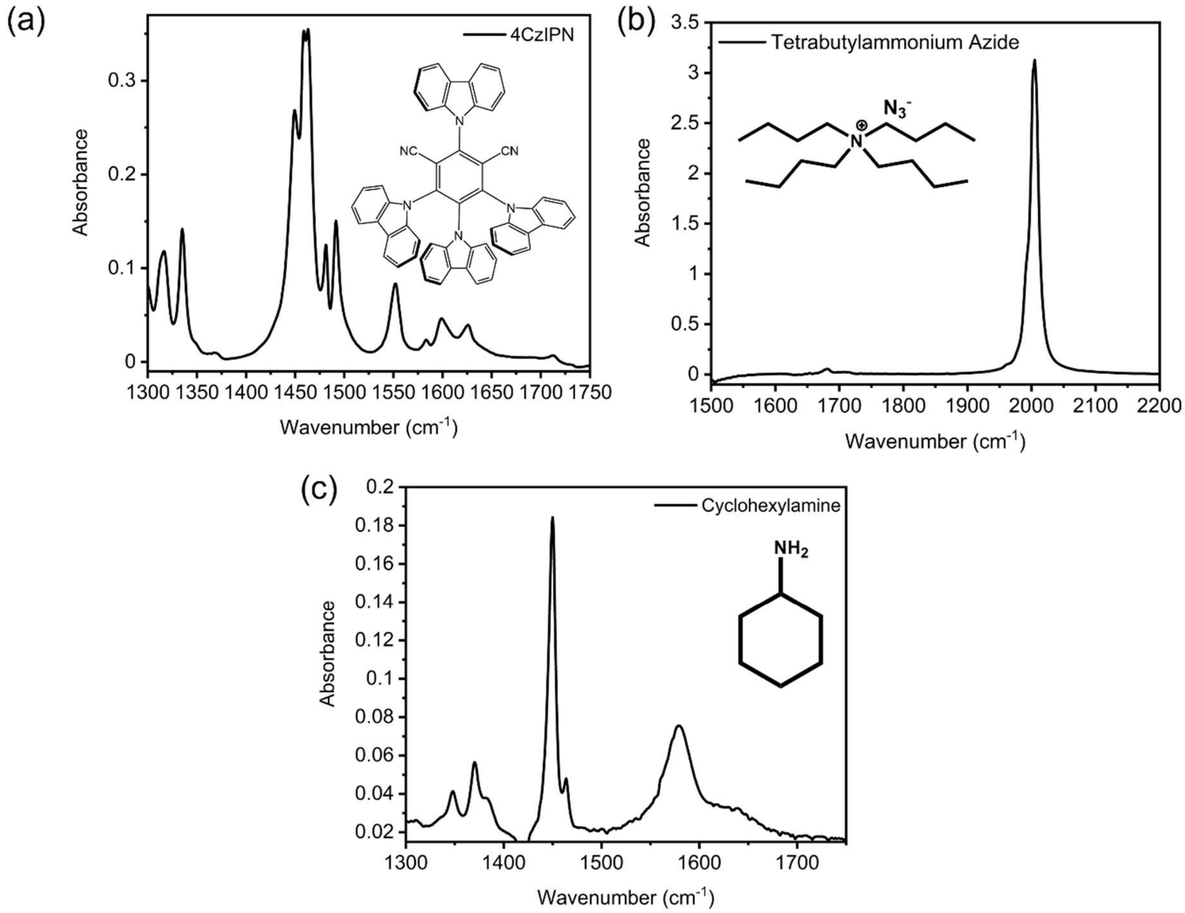

Fig. S2 *Steady state FTIR spectra of solutions of: (a) 10 mM 4CzIPN; (b) 25 mM TBAA; and (c) 100 mM CHA. Measurements for 4CzIPN and CHA used a cell with a 200-µm sample pathlength, whereas TBAA measurements used a 300-µm cell. 4CzIPN and TBAA solutions were prepared in MeCN-d$_3$, with the CHA solution prepared in dichloromethane.*

Section S2: Computational Methodology

The interpretation of experimental transient absorption spectra made use of predictions from DFT calculations[36,37] to support the assignment of spectral bands in TEAS or TVAS data. All DFT calculations used the Gaussian 09W software package.[8] These methods are described in greater detail here.

Frequency optimization calculations were performed on all reactive intermediates using a 6-31G++(d,p) basis set with a B3LYP or PBE functional.[9-11] An acetonitrile IEFPCM solvent model[12,13] was applied to represent the experimental solution. Calculations performed on 4CzIPN instead used a 6-31G(d) basis set because of its structural complexity. The calculations provided optimized structures and energies, as well as predictions of vibrational frequencies. For stable compounds, comparison of computed IR band wavenumbers with steady-state FTIR spectra established a scaling factor to account for vibrational anharmonicity which was then applied to all DFT frequency optimized calculations for that molecule and its corresponding reaction intermediates. A scaling factor of 0.985 was obtained for 4CzIPN, 0.98 for TBAA, and 0.95 for CHA. These corrections were derived using measured vibrational bands at 1463 cm^{-1}, 2005 cm^{-1} and 2927 cm^{-1} and their corresponding computed bands at 1485 cm^{-1}, 2055 cm^{-1} and 3080 cm^{-1} for 4CzIPN, TBAA and CHA respectively.

Where required, time-dependent DFT (TD-DFT) calculations were performed to predict the UV-Vis absorption spectra of molecules and intermediates, assign their electronic transitions, and compute oscillator strengths. All TD-DFT calculations were performed from the corresponding geometry-optimized ground-state structures with the same level of theory and method of solvent representation, but with CAM-B3LYP used for 4CzIPN.

Possible mechanisms of the HAT reactions were investigated using quantum mechanical calculations performed with Gaussian 16 (Revision C.01)[14] to explore the roles of $N_3^{\bullet}$ and $N_6^{\bullet -}$ radicals (*vide infra*). All geometries were optimized using the M06-2X density functional with the 6-31G(d) basis set within the IEFPCM (acetonitrile or THF) model. All single point energies were calculated using M06-2X and the 6-311G(d,p) basis set within the IEFPCM (acetonitrile or THF) model. The resulting energies were used to correct the energies obtained from the M06-2X

optimizations.[15] For HAT $N_3^•$ reactions with THF, six conformers for each possible reaction centre were considered. These conformers in every case converged to one of two possible conformers for each specific reaction centre. β-O HAT was calculated to be strongly disfavoured relative to α-O HAT for THF in acetonitrile and THF by 31.1 and 31.3 kJ mol^{-1} respectively. All temperature (298.15 K) and concentration-corrected (0.15 M) quasi-harmonic (Grimme approximation) Gibbs energies were calculated with GoodVibes[16] with a vibrational scaling factor of 1.0. All computed structures were illustrated with CYLView.[17] Section S5 of Supporting Information reports the solvent models used and the detailed outcomes from these calculations.

Section S3 Comparison of Measured and Computed Spectra

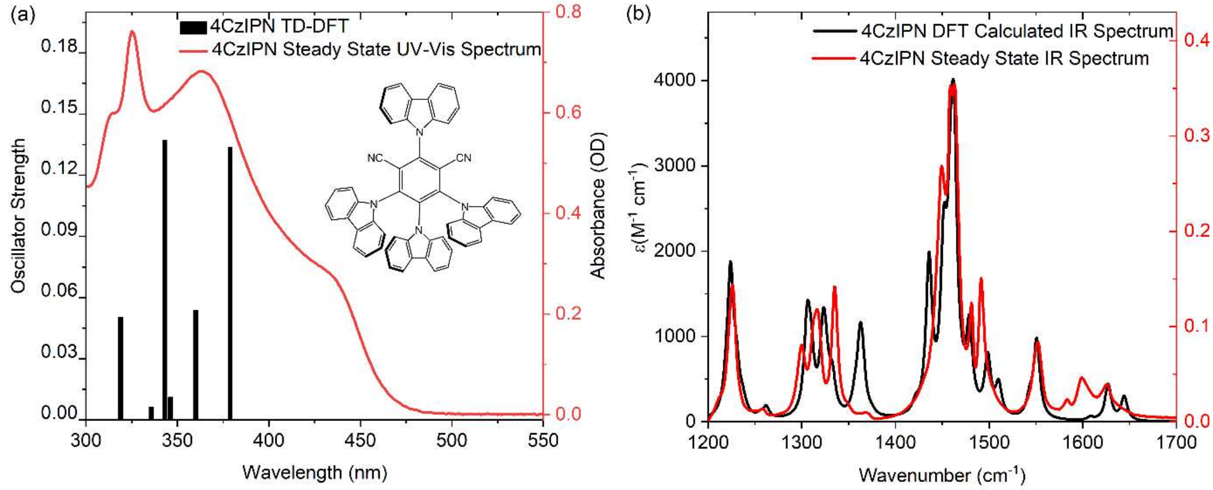

Fig. S3 *(a) Comparison of the steady-state UV-Vis spectrum of 2.5 mM 4CzIPN in MeCN (red line), obtained with a 300-μm sample pathlength, and a computed TD-DFT spectrum of 4CzIPN with an IEFPCM acetonitrile solvent model (black bars). The calculation was performed at the CAM-B3LYP/6-31G(d) level of theory. (b) Comparison of the steady state IR spectrum of 10 mM 4CzIPN in MeCN-d_3 (red line, 200-μm sample pathlength) and the DFT calculated IR spectrum of 4CzIPN (black line) obtained at the 6-31G(d) level of theory with an IEFPCM acetonitrile solvent model. Computed harmonic IR band wavenumbers have been corrected by a scaling factor of 0.985.*

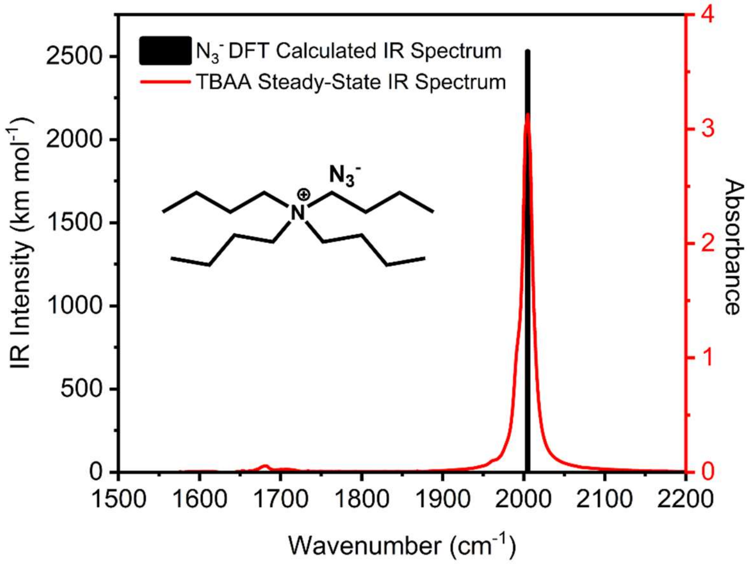

Fig. S4 *Comparison of the steady-state IR spectrum of 25 mM TBAA in MeCN-d_3 (red line), obtained with a 300-μm sample pathlength, and the DFT calculated IR spectrum of N_3^- (black bar). The calculation was performed at the B3LYP/6-31G++(d,p) level of theory and used an IEFPCM acetonitrile solvent model. Computed harmonic IR band wavenumbers have been corrected by a scaling factor of 0.98.*

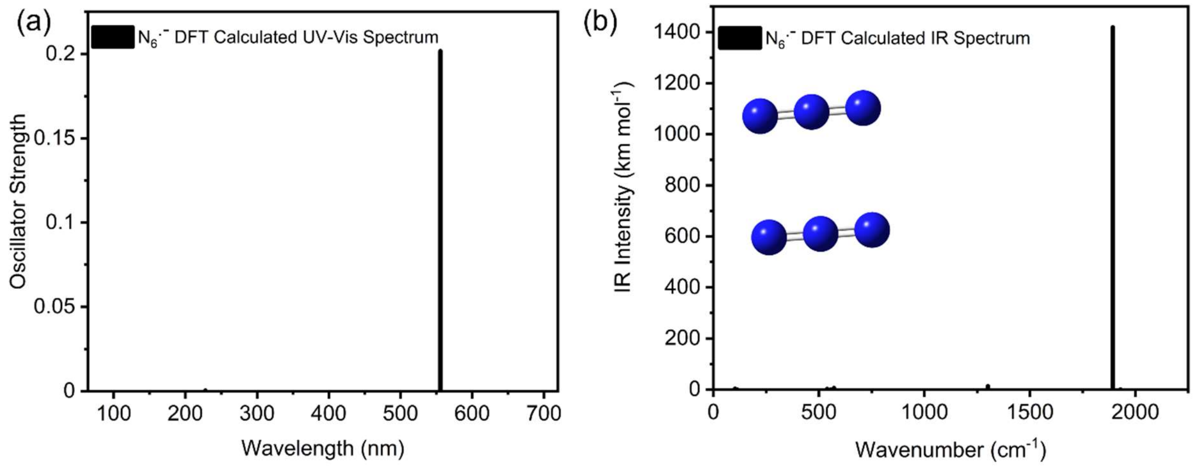

Fig. S5 *(a) TD-DFT calculated UV-Vis spectrum of $N_6^{\bullet -}$; (b) DFT calculated IR spectrum of $N_6^{\bullet -}$. In both calculations, an optimized cyclic geometry was obtained for $N_6^{\bullet -}$ as shown in the inset to (b). The calculations included an IEFPCM acetonitrile solvent model and were performed at the PBE0/6-31G++(d,p) level of theory. Computed harmonic IR band wavenumbers have been corrected by a scaling factor of 0.98.*

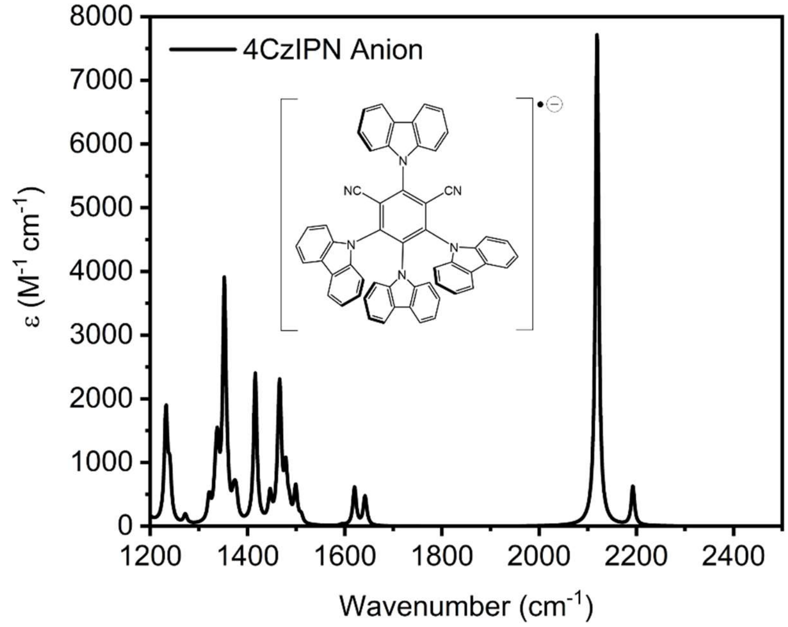

Fig. S6 *DFT calculated IR spectrum of the radical anion of 4CzIPN with an IEFPCM acetonitrile solvent model, performed at the B3LYP/6-31G level of theory. Computed harmonic IR band wavenumbers have been corrected by a scaling factor of 0.98.*

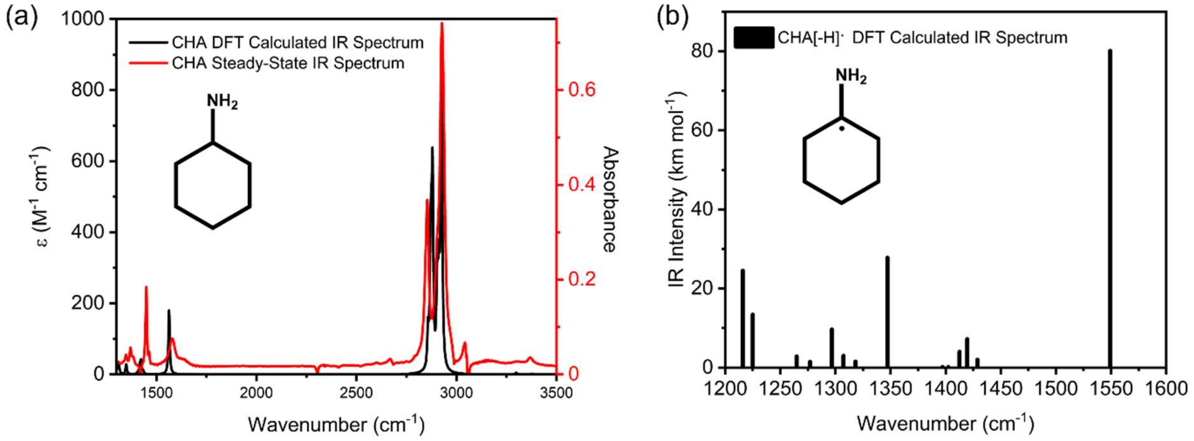

Fig S7 *(a) Comparison of the steady-state IR spectrum of 100 mM CHA in dichloromethane (red line), obtained with a 200-μm sample pathlength, and the DFT calculated IR spectrum of CHA (black line). The calculation was performed at the B3LYP/6-31G++(d,p) level of theory with an IEFPCM acetonitrile solvent model. (b) DFT calculated IR spectrum of the CHA[-H]$^{\bullet}$ radical, performed at the 6-31G++(d,p) level of theory with an IEFPCM acetonitrile solvent model. Computed harmonic IR band wavenumbers have been corrected by a scaling factor of 0.95.*

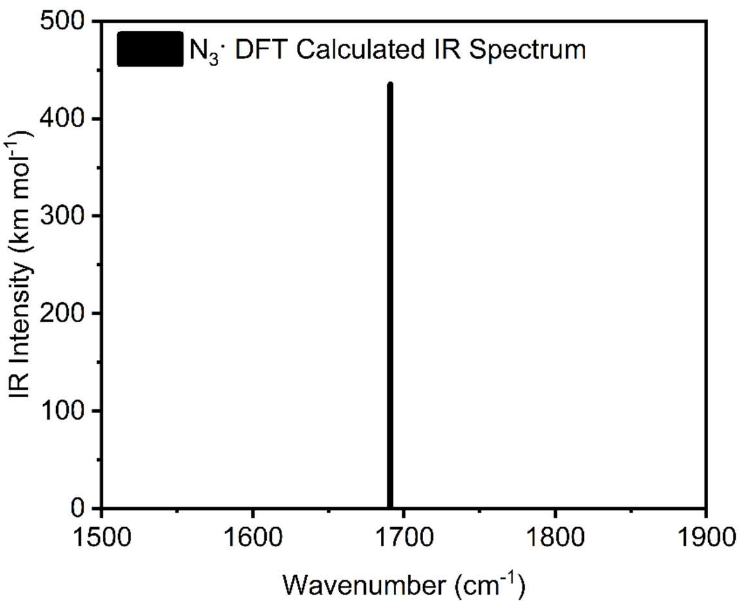

Fig S8 *DFT calculated IR spectrum of $N_3^{\bullet}$, performed at the B3LYP/6-31G++(d,p) level of theory with an IEFPCM acetonitrile solvent model. Computed harmonic IR band wavenumbers have been corrected by a scaling factor of 0.98.*

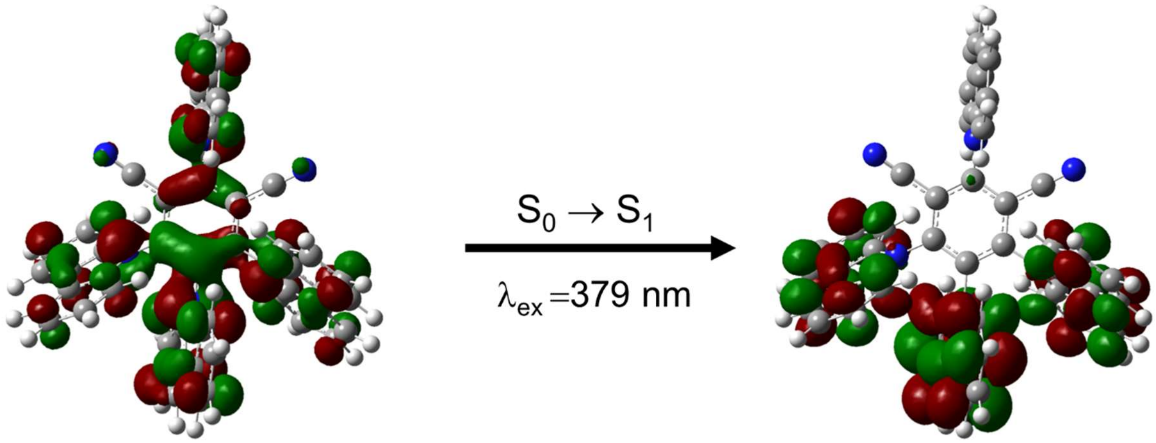

Fig S9 *Orbital diagrams for the $S_1 \leftarrow S_0$ transition in 4CzIPN. Only HOMO to LUMO transition is shown. The TD-DFT calculation used the CAM-B3LYP/6-31G (d) level of theory with an acetonitrile IEFPCM solvent model.*

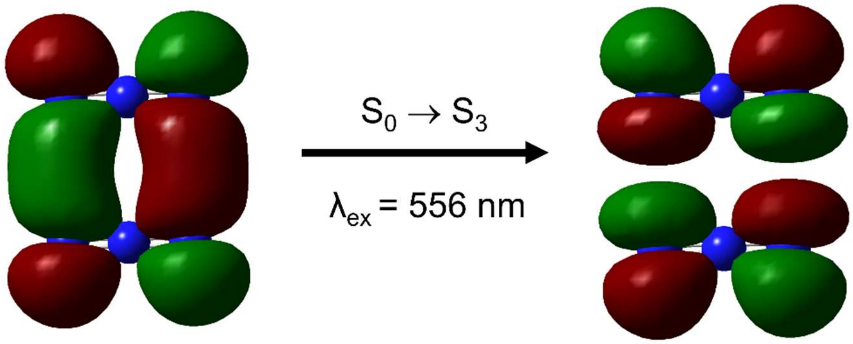

Fig S10 *Orbital diagrams for the $S_3 \leftarrow S_0$ transition in $N_6^{\bullet -}$. This transition is the only one computed to lie in the visible region of the spectrum and therefore to contribute to the experimental TEAS spectra. The TD-DFT calculation used the B3LYP/6-31++G(d,p) level of theory with an acetonitrile IEFPCM solvent model.*

Section S4 Decomposition of Transient Absorption Spectra and Extracted Kinetic Data

Table S1. *Extracted kinetic data from TVAS experimental measurements for 4CzIPN in MeCN-d_3* photoexcited at 425 nm.

Concentration of 4CzIPN (mM)	Wavenumber of peak (cm^{-1})	Assignment	τ_2 (ns)	τ_3 (ns)
2.5	1370-1430	4CzIPN* (S_1)	21.5 ± 1.0	1500 ± 170
2.5	1440-1500	4CzIPN (S_0)	21.5 ± 1.0	1500 ± 170
2.5	1565-1755	4CzIPN* (S_1)	21.5	1500

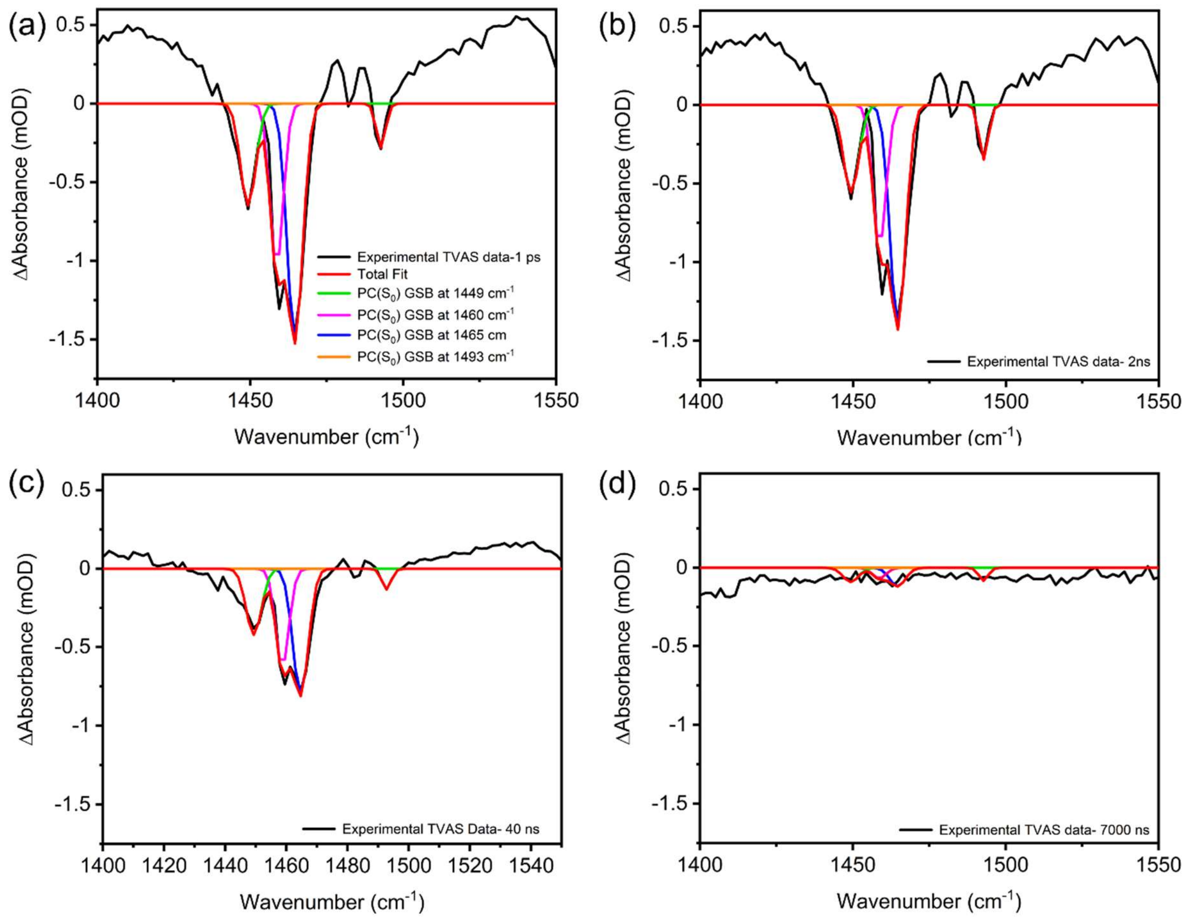

Fig. S11 *Decomposition of TVAS data using the KOALA program*[1] *for a solution of 2.5 mM 4CzIPN in MeCN-d_3. The variations in band intensities were fitted with an integration window over the range 1370-1425cm^{-1} to capture the decay of 4CzIPN*(S_1) and 4 Gaussian functions to describe the loss of the ground-state bleach features corresponding to depopulation of the 4CzIPN(S_0) state. The example decompositions shown here focus on the ground-state bleach features.*

***Table S2** Time constants and bimolecular rate coefficients for electron transfer extracted from TVAS measurements for solutions of 4CzIPN and TBAA in MeCN-d_3 in which the concentration of TBAA ranged from 8 – 40 mM.*

Concentration of 4CzIPN (mM)	Concentration of Azide (mM)	Wavenumber of Peak (cm^{-1})	Assignment	τ_1 (ns)	τ_2 (ns)	τ_a (ns)	τ_b (ns)	k_a / (10^{10} M^{-1} s^{-1})
2.5	8	1735-1925	4CzIPN* (S_1)	0.035 ± 0.004	4.8 ± 0.2		-	2.4 ± 0.2
2.5	8	1829	$N_6^{\bullet-}$	-	-	4.8	8.4 ± 0.9	-
2.5	8	1930-2215	4CzIPN* (S_1)	-	4.8		-	-
2.5	8	2125	$4CzIPN^-$	-	4.8		-	-
2.5	8	2005	$N_3^\bullet$	-	-	4.8	8.4	-
2.5	16	1735-1920	4CzIPN* (S_1)	0.041 ± 0.005	2.7 ± 0.1		-	2.4 ± 0.2
2.5	16	1829	$N_6^{\bullet-}$	-	-	2.7	4.2 ± 0.4	-
2.5	16	1935-2210	4CzIPN* (S_1)	-	2.7		-	-
2.5	16	2125	$4CzIPN^-$	-	2.7		-	-
2.5	16	2005	$N_3^\bullet$	-	-	2.7	4.2	-
2.4	24	1740-1920	4CzIPN* (S_1)	0.019 ± 0.003	1.68 ± 0.08		-	2.4 ± 0.2
2.4	24	1829	$N_6^{\bullet-}$	-	-	1.68	2.7 ± 0.2	-
2.4	24	1940-2200	4CzIPN* (S_1)	-	1.68		-	-
2.4	24	2125	$4CzIPN^-$	-	1.68		-	-
2.4	24	2005	$N_3^\bullet$	-	-	1.68	2.7	-
2.4	32	1735-1905	4CzIPN* (S_1)	0.052 ± 0.008	1.41 ± 0.08		-	2.4 ± 0.2
2.4	32	1829	$N_6^{\bullet-}$	-	-	1.41	1.85 ± 0.10	-
2.4	32	1935-2220	4CzIPN* (S_1)	-	1.41		-	-
2.4	32	2125	$4CzIPN^-$	-	1.41		-	-
2.4	32	2005	$N_3^\bullet$	-	-	1.41	1.85	-
2.4	40	1740-1920	4CzIPN* (S_1)	0.027 ± 0.004	1.01 ± 0.05		-	2.4 ± 0.2
2.4	40	1829	$N_6^{\bullet-}$	-	-	1.01	1.6 ± 0.1	-
2.4	40	1945-2210	4CzIPN* (S_1)	-	1.01		-	-
2.4	40	2125	$4CzIPN^-$	-	1.01		-	-
2.4	40	2005	$N_3^\bullet$	-	-	1.01	1.6	-

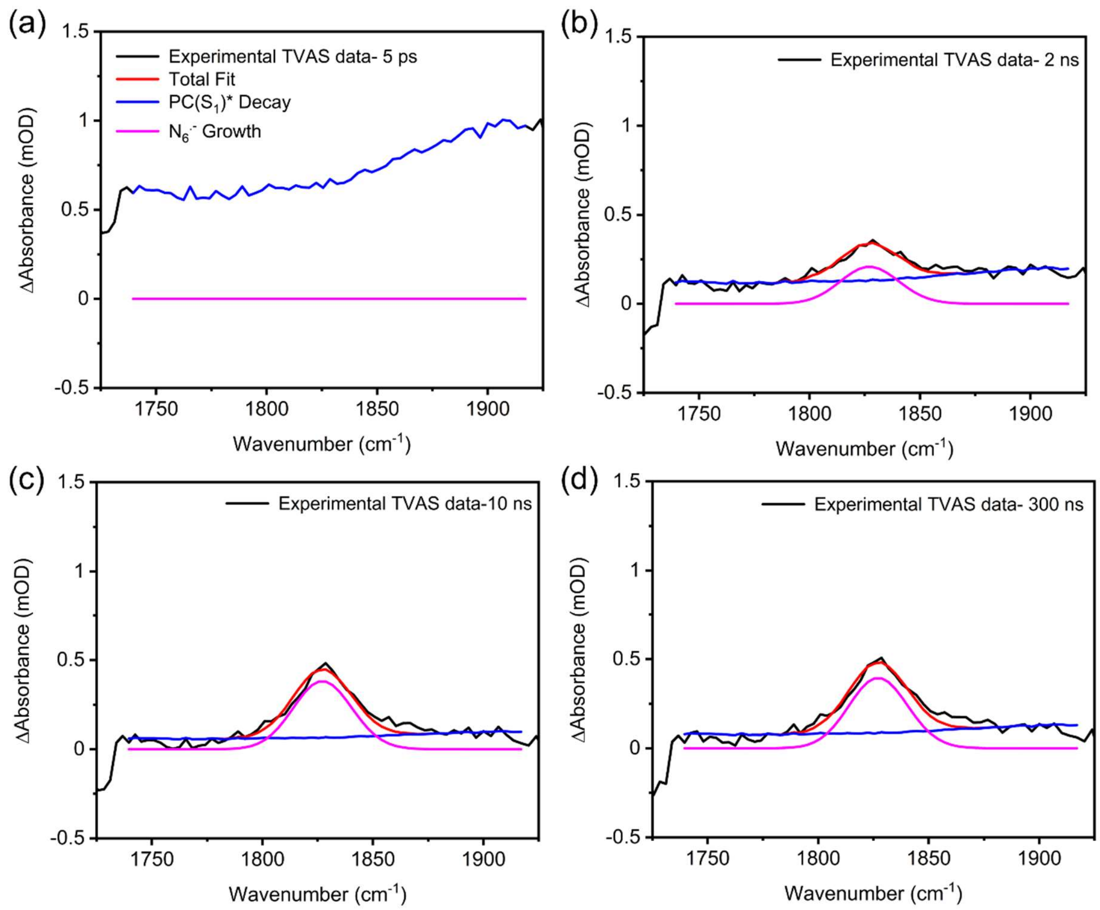

Fig. S12 *Decomposition of TVAS data using the KOALA program*[1] *for a solution of 2.4 mM 4CzIPN with 40 mM TBAA in MeCN-d_3. The variations in band intensities were fitted with an early time (5 ps) basis function (blue line) to capture the decay of 4CzIPN* (S_1) and an overlayed Gaussian function (pink line) to account for the growth of an $N_6^{\bullet-}$ absorption band.*

Table S3 *Time constants and bimolecular rate coefficients for H atom transfer, obtained from TVAS measurements for solutions of 4CzIPN, TBAA and CHA in MeCN-d_3 in which the concentrations of 4CzIPN, TBAA and CHA ranged from 1.6-1.8 mM, 17-19 mM and 250-920 mM respectively.*

Concentration of 4CzIPN (mM)	Concentration of TBAA (mM)	Concentration of CHA (mM)	Wavenumber of Peak (cm^{-1})	Assignment	τ_1 (ns)	τ_2 (ns)	τ_c (ns)	τ_d (ns)	k_c / (10^6 M^{-1} s^{-1})
1.8	19	250	1800-1975	4CzIPN* (S_1)	0.04 ± 0.02	3.4 ± 0.1	-	-	-
1.8	19	250	1840	$N_6^{\cdot-}$	-	-	934 ± 158		2.2 ± 0.3
1.8	19	250	2018	4CzIPN* (S_1)	0.04	3.4	-	-	-
1.8	19	250	2018	CHA[-H]$^{\cdot}$ + O_2	-	-	-	1438 ± 181	-
1.7	18	486	1800-1975	4CzIPN* (S_1)	0.005 ± 0.003	3.1 ± 0.1	-	-	-
1.7	18	486	1840	$N_6^{\cdot-}$	-	-	738 ± 89	-	2.2 ± 0.3
1.7	18	486	2018	4CzIPN* (S_1)	0.005	3.1	-	-	-
1.7	18	486	2018	CHA[-H]$^{\cdot}$ + O_2	-	-	-	882 ± 82	-
1.7	18	709	1800-1965	4CzIPN* (S_1)	0.018 ± 0.010	3.2 ± 0.1			-
1.7	18	709	1837	$N_6^{\cdot-}$	-	-	523 ± 61	-	2.2 ± 0.3
1.7	18	709	2014	4CzIPN* (S_1)	0.018	3.2	-	-	-
1.7	18	709	2014	CHA[-H]$^{\cdot}$ + O_2	-	-	-	669 ± 58	-
1.6	17	920	1810-1960	4CzIPN* (S_1)	0.006 ± 0.003	3.1 ± 0.1			-
1.6	17	920	1840	$N_6^{\cdot-}$	-	-	395 ± 101	-	2.2 ± 0.3
1.6	17	920	2014	4CzIPN* (S_1)	0.006	3.1			-
1.6	17	920	2014	CHA[-H]$^{\cdot}$ + O_2	-	-	-	532 ± 46	-

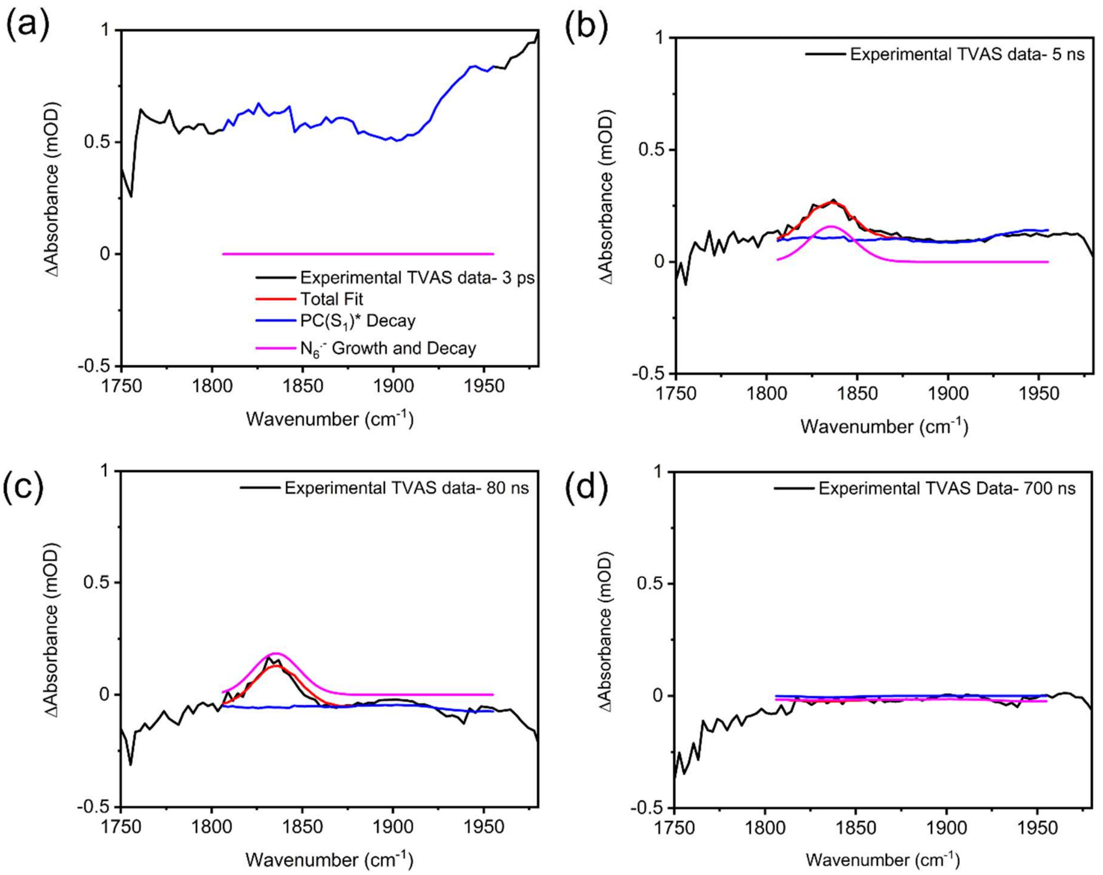

Fig. S13 *Decomposition of TVAS data using the KOALA program*[1] *for a solution of 1.6 mM 4CzIPN with 17 mM TBAA and 920 mM CHA in MeCN-d_3. The variations in band intensities were fitted with an early time (3 ps) basis function (pink line) to describe the decay of 4CzIPN*(S_1) and an overlayed Gaussian function (blue line) to account for the growth and decay of $N_6^{\cdot -}$.*

Table S4 *Time constants for 4CzIPN relaxation extracted from TEAS measurements for a solution of 2.5 mM 4CzIPN in MeCN.*

Concentration of 4CzIPN (mM)	Wavelength of peak (nm)	Assignment	τ (ns)	τ (ns)
2.5	470	4CzIPN* (S_1)	0.0010 ± 0.0001	0.025 ± 0.002
2.5	600-750	4CzIPN* (S_n) or vibrationally excited 4CzIPN* (S_1)	0.0010 ± 0.0001	0.025 ± 0.002

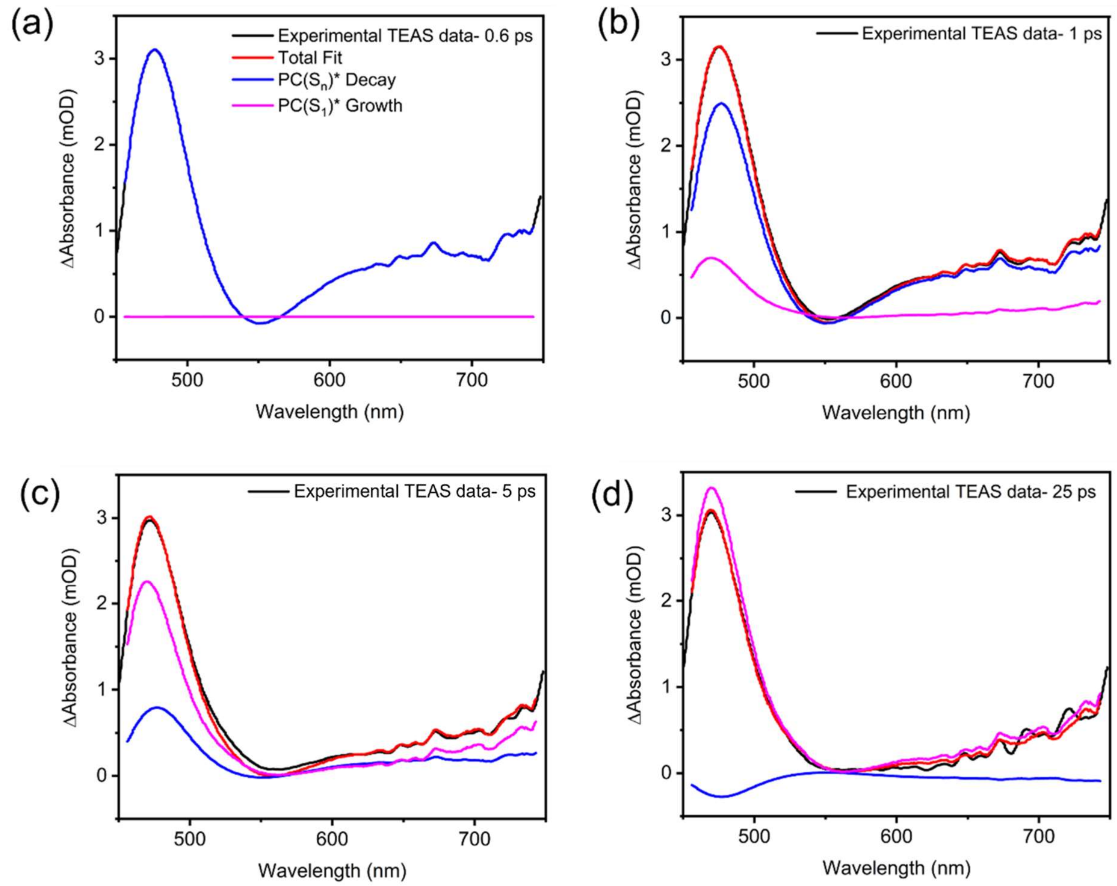

Fig. S14 *Decomposition of TEAS data using the KOALA program*[1] *for a solution of 2.5 mM 4CzIPN in MeCN. The variations in band intensities were fitted with an early-time (0.6 ps) basis function (blue line) to describe the decay of 4CzIPN*(S_n) and a late-time basis function (1338 ps) (pink line) to account for the growth of 4CzIPN*(S_1).*

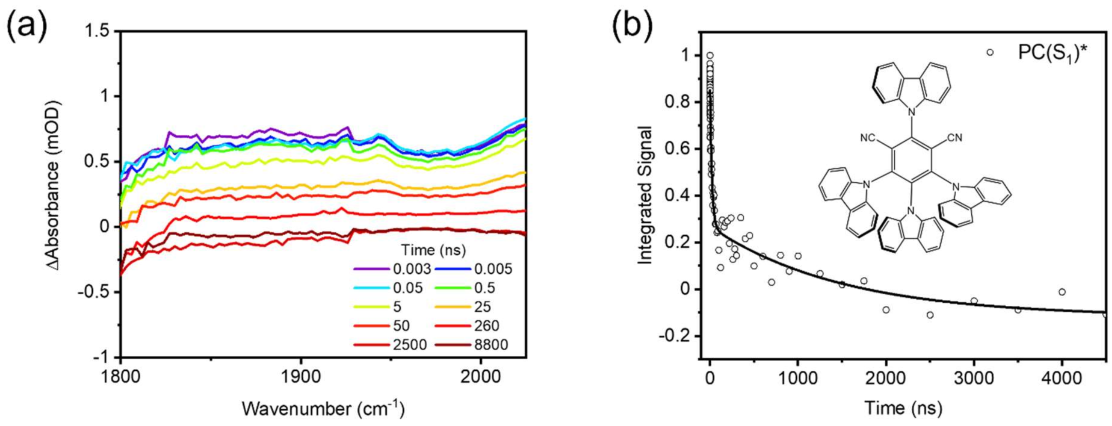

Fig S15 *Transient absorption spectra obtained for a 425-nm photoexcited 4CzIPN solution in MeCN-d$_3$ at probe wavenumbers above 1800 cm^{-1}, and the derived kinetic trace. (a) TVAS spectra of 2.5-mM 4CzIPN in MeCN-d$_3$; (b) kinetic trace obtained from analysis of the broad band intensity in the TVAS spectrum in (a), and a biexponential fit. The basis function used for spectral decomposition was selected to be an early time spectrum representing the excited state absorption from the PC(S$_1$)* state. The derived kinetics therefore account for decay of the PC(S$_1$)* state population.*

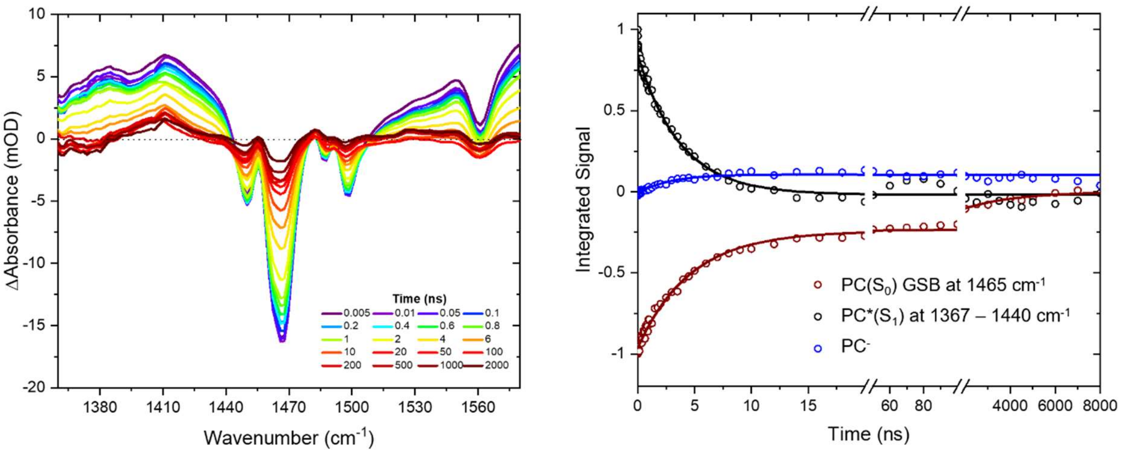

Fig S16 *Left: Transient absorption spectra obtained for a 425-nm photoexcited 2.5 mM 4CzIPN and 8 mM TBAA solution in MeCN-d$_3$ at probe wavenumbers from 1360 – 1580 cm^{-1}. Right: Decay of the integrated band intensities of features corresponding to 4CzIPN PC*(S$_1$) excited state absorption (black circles) and PC(S$_0$) ground-state bleach (brown circles), and growth of the weak PC$^{\bullet-}$ radical anion absorption feature at 1410 cm^{-1} (blue circles). Solid lines are fits to bi-exponential decays, with the second time constant for the ESA and PC$^{\bullet-}$ decay globally fitted to the same value. For the ESA decay,* $\tau_1 = 0.003 \pm 0.001$ ns, $\tau_2 = 3.70 \pm 0.09$ ns (with the same value for *PC$^{\bullet-}$ growth). For the GSB recovery,* $\tau_1 = 4.58 \pm 0.12$ ns, $\tau_2 = 2290 \pm 330$ ns.

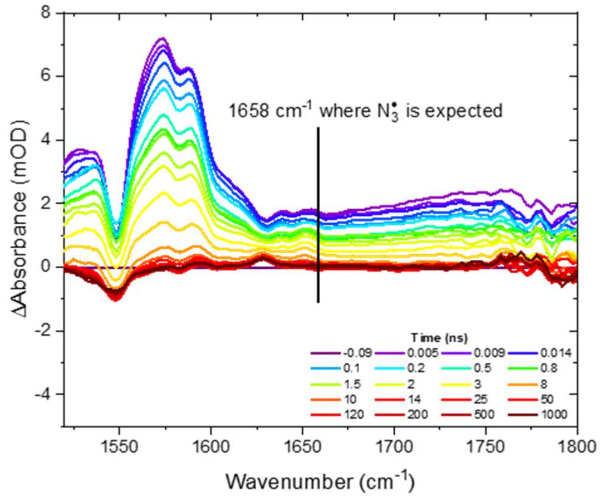

Fig S17 *Left: Transient absorption spectra obtained for a 425-nm photoexcited 2.5 mM 4CzIPN and 16 mM TBAA solution in MeCN-d$_3$ at probe wavenumbers from 1520 – 1800 cm^{-1}. An absorption band of the N$_3^\bullet$ radical expected at 1658 cm^{-1} is not seen. The broad ESA features observed across this spectral window correspond to 4CzIPN PC*(S$_1$) ESA, as also seen in Fig S16. The weak feature at 1620 cm^{-1} is assigned to the 4CzIPN PC$^{\bullet-}$ radical anion.*

Section S5 Computational Data

All reported energies in Hartrees. All reported frequencies are in cm^{-1}.

N_3^- in Acetonitrile

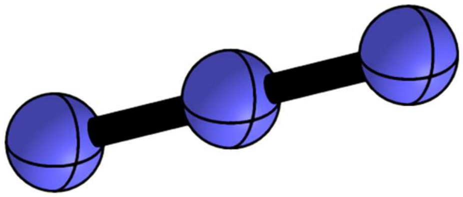

M06-2X/6-31G(d)-IEFPCM(acetonitrile) Energy	-164.245451
M06-2X/6-311G(d,p)-IEFPCM(acetonitrile) Energy	-164.294155
M06-2X/6-311G(d,p)-IEFPCM(acetonitrile) Free Energy (Quasiharmonic)	-164.30199
Number of Imaginary Frequencies	0

M06-2X/6-31G(d)-IEFPCM(acetonitrile) Molecular Geometry in Cartesian Coordinates

N	0.000000	0.000000	1.177856
N	0.000000	0.000000	0.000000
N	0.000000	0.000000	-1.177856

$N_3^{\bullet}$ in Acetonitrile

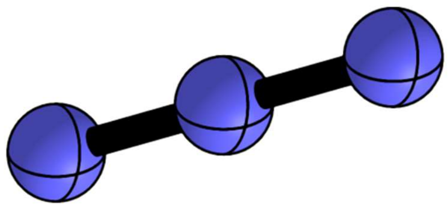

M06-2X/6-31G(d)-IEFPCM(acetonitrile) Energy	-164.067397
M06-2X/6-311G(d,p)-IEFPCM(acetonitrile) Energy	-164.110305
M06-2X/6-311G(d,p)-IEFPCM(acetonitrile) Free Energy (Quasiharmonic)	-164.120731
Number of Imaginary Frequencies	0

M06-2X/6-31G(d)-IEFPCM(acetonitrile) Molecular Geometry in Cartesian Coordinates

N	-0.000000	0.000000	1.174876
N	0.000000	0.000000	0.000000
N	0.000000	-0.000000	-1.174876

$N_6^{\bullet -}$ Structure 1 in Acetonitrile

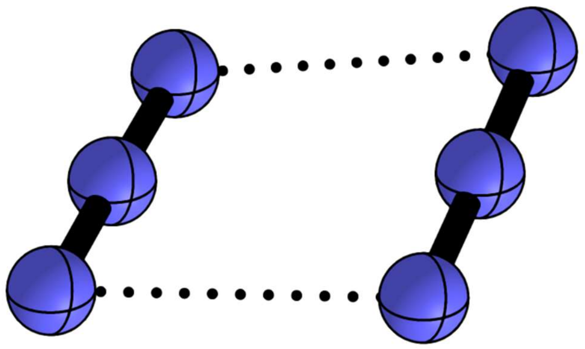

M06-2X/6-31G(d)-IEFPCM(acetonitrile) Energy	-328.341012
M06-2X/6-311G(d,p)-IEFPCM(acetonitrile) Energy	-328.430967
M06-2X/6-311G(d,p)-IEFPCM(acetonitrile) Free Energy (Quasiharmonic)	-328.437549
Number of Imaginary Frequencies	0

M06-2X/6-31G(d)-IEFPCM(acetonitrile) Molecular Geometry in Cartesian Coordinates

N	1.327072	1.174343	0.056881
N	1.337831	0.000081	0.000003
N	1.327471	-1.174186	-0.056878
N	-1.327341	1.174187	-0.056881
N	-1.337832	-0.000083	-0.000003
N	-1.327202	-1.174341	0.056878

$N_6^{\bullet-}$ Structure 2 in Acetonitrile

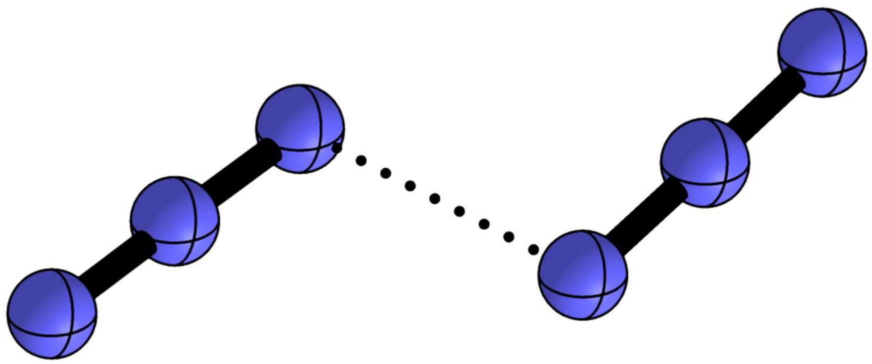

M06-2X/6-31G(d)-IEFPCM(acetonitrile) Energy	-328.333131
M06-2X/6-311G(d,p)-IEFPCM(acetonitrile) Energy	-328.423527
M06-2X/6-311G(d,p)-IEFPCM(acetonitrile) Free Energy (Quasiharmonic)	-328.43023
Number of Imaginary Frequencies	0

M06-2X/6-31G(d)-IEFPCM(acetonitrile) Molecular Geometry in Cartesian Coordinates

N	0.732768	0.884251	0.094131
N	1.728087	0.243836	-0.000491
N	2.697584	-0.396399	-0.093641
N	-2.697584	0.396399	-0.093641
N	-1.728087	-0.243836	-0.000490
N	-0.732768	-0.884251	0.094133

$N_6^{\bullet-}$ Structure 3 in Acetonitrile

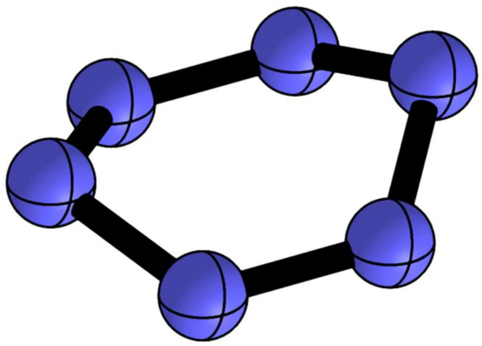

M06-2X/6-31G(d)-IEFPCM(acetonitrile) Energy	-328.317685
M06-2X/6-311G(d,p)-IEFPCM(acetonitrile) Energy	-328.400104
M06-2X/6-311G(d,p)-IEFPCM(acetonitrile) Free Energy (Quasiharmonic)	-328.402876
Number of Imaginary Frequencies	0

M06-2X/6-31G(d)-IEFPCM(acetonitrile) Molecular Geometry in Cartesian Coordinates

N	1.130710	0.636684	-0.118805
N	-0.000473	1.306765	0.237548
N	-1.131179	0.635869	-0.118768
N	-1.130708	-0.636689	-0.118755
N	0.000494	-1.306760	0.237504
N	1.131156	-0.635870	-0.118724

$N_6^{\bullet-}$ Structure 4 in Acetonitrile

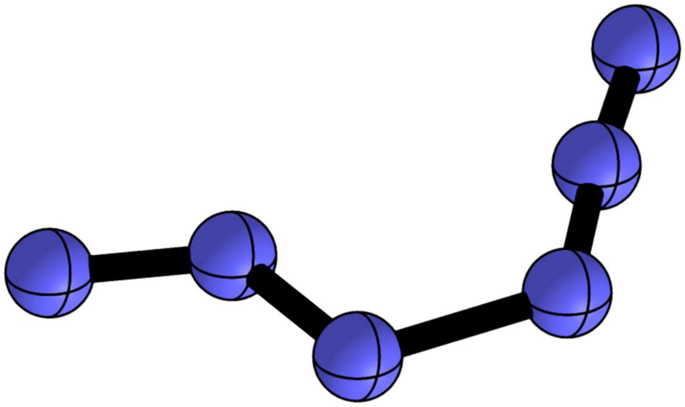

M06-2X/6-31G(d)-IEFPCM(acetonitrile) Energy	-328.299843
M06-2X/6-311G(d,p)-IEFPCM(acetonitrile) Energy	-328.387915
M06-2X/6-311G(d,p)-IEFPCM(acetonitrile) Free Energy (Quasiharmonic)	-328.394131
Number of Imaginary Frequencies	0

M06-2X/6-31G(d)-IEFPCM(acetonitrile) Molecular Geometry in Cartesian Coordinates

N	0.728187	1.031516	0.000319
N	1.346065	-0.027002	0.000006
N	1.994755	-0.971284	-0.000144
N	-2.246356	-0.701154	-0.000007
N	-1.111216	-0.307233	0.000236
N	-0.711435	0.975156	-0.000411

Tetrahydrofuran in Acetonitrile

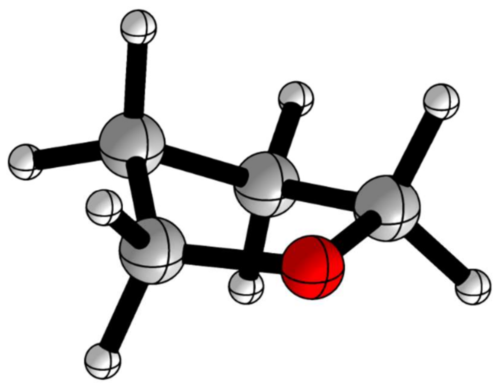

M06-2X/6-31G(d)-IEFPCM(acetonitrile) Energy	-232.340223
M06-2X/6-311G(d,p)-IEFPCM(acetonitrile) Energy	-232.406182
M06-2X/6-311G(d,p)-IEFPCM(acetonitrile) Free Energy (Quasiharmonic)	-232.314137
Number of Imaginary Frequencies	0

M06-2X/6-31G(d)-IEFPCM(acetonitrile) Molecular Geometry in Cartesian Coordinates

C	1.160987	-0.425665	0.136823
O	0.000002	-1.246137	0.000022
C	-1.160978	-0.425672	-0.136851
C	-0.727244	0.990045	0.237571
C	0.727231	0.990058	-0.237559
H	1.512791	-0.464539	1.176803
H	1.950017	-0.822137	-0.509287
H	-1.512718	-0.464532	-1.176854
H	-1.950044	-0.822159	0.509204
H	-1.343533	1.757825	-0.234807
H	-0.770406	1.127079	1.323086
H	0.770387	1.127126	-1.323069
H	1.343514	1.757831	0.234839

N_3^- in THF

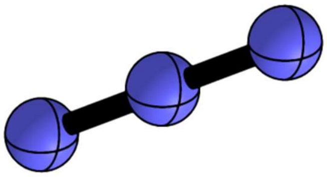

M06-2X/6-31G(d)-IEFPCM(THF) Energy	-164.234387
M06-2X/6-311G(d,p)-IEFPCM(THF) Energy	-164.283093
M06-2X/6-311G(d,p)-IEFPCM(THF) Free Energy (Quasiharmonic)	-164.290944
Number of Imaginary Frequencies	0

M06-2X/6-31G(d)-IEFPCM(THF) Molecular Geometry in Cartesian Coordinates

N	0.000000	0.000000	1.177856
N	0.000000	0.000000	0.000000
N	0.000000	0.000000	-1.177856

$N_3^{\bullet}$ in THF

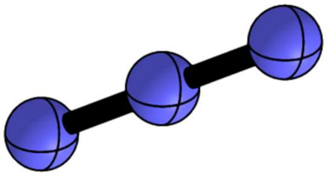

M06-2X/6-31G(d)-IEFPCM(THF) Energy	-164.067016
M06-2X/6-311G(d,p)-IEFPCM(THF) Energy	-164.109879
M06-2X/6-311G(d,p)-IEFPCM(THF) Free Energy (Quasiharmonic)	-164.120307
Number of Imaginary Frequencies	0

M06-2X/6-31G(d)-IEFPCM(THF) Molecular Geometry in Cartesian Coordinates

N	-0.000000	0.000000	1.174876
N	0.000000	0.000000	0.000000
N	0.000000	-0.000000	-1.174876

$N_6^{\bullet-}$ Structure 1 in THF

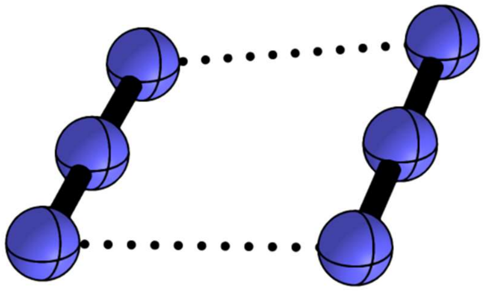

M06-2X/6-31G(d)-IEFPCM(THF) Energy	-328.331574
M06-2X/6-311G(d,p)-IEFPCM(THF) Energy	-328.421496
M06-2X/6-311G(d,p)-IEFPCM(THF) Free Energy (Quasiharmonic)	-328.427845
Number of Imaginary Frequencies	0

M06-2X/6-31G(d)-IEFPCM(THF) Molecular Geometry in Cartesian Coordinates

N	1.327072	1.174343	0.056881
N	1.337831	0.000081	0.000003
N	1.327471	-1.174186	-0.056878
N	-1.327341	1.174187	-0.056881
N	-1.337832	-0.000083	-0.000003
N	-1.327202	-1.174341	0.056878

$N_6^{\bullet-}$ Structure 2 in THF

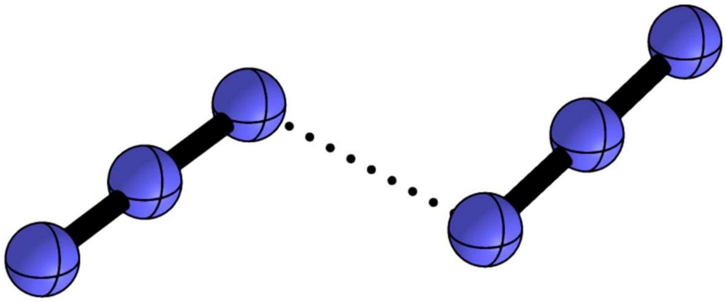

M06-2X/6-31G(d)-IEFPCM(THF) Energy	-328.324147
M06-2X/6-311G(d,p)-IEFPCM(THF) Energy	-328.414549
M06-2X/6-311G(d,p)-IEFPCM(THF) Free Energy (Quasiharmonic)	-328.421093
Number of Imaginary Frequencies	0

M06-2X/6-31G(d)-IEFPCM(THF) Molecular Geometry in Cartesian Coordinates

N	0.732768	0.884251	0.094131
N	1.728087	0.243836	-0.000491
N	2.697584	-0.396399	-0.093641
N	-2.697584	0.396399	-0.093641
N	-1.728087	-0.243836	-0.000490
N	-0.732768	-0.884251	0.094133

$N_6^{\bullet-}$ Structure 3 in THF

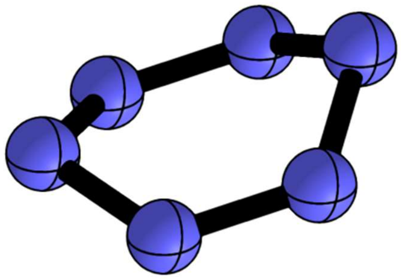

M06-2X/6-31G(d)-IEFPCM(THF) Energy	-328.308131
M06-2X/6-311G(d,p)-IEFPCM(THF) Energy	-328.390517
M06-2X/6-311G(d,p)-IEFPCM(THF) Free Energy (Quasiharmonic)	-328.393307
Number of Imaginary Frequencies	0

M06-2X/6-31G(d)-IEFPCM(THF) Molecular Geometry in Cartesian Coordinates

N	1.130710	0.636684	-0.118805
N	-0.000473	1.306765	0.237548
N	-1.131179	0.635869	-0.118768
N	-1.130708	-0.636689	-0.118755
N	0.000494	-1.306760	0.237504
N	1.131156	-0.635870	-0.118724

$N_6^{\bullet-}$ Structure 4 in THF

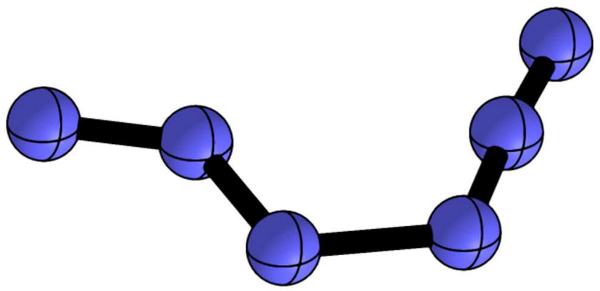

M06-2X/6-31G(d)-IEFPCM(THF) Energy	-328.2903
M06-2X/6-311G(d,p)-IEFPCM(THF) Energy	-328.37834
M06-2X/6-311G(d,p)-IEFPCM(THF) Free Energy (Quasiharmonic)	-328.38464
Number of Imaginary Frequencies	0

M06-2X/6-31G(d)-IEFPCM(THF) Molecular Geometry in Cartesian Coordinates

N	0.728187	1.031516	0.000319
N	1.346065	-0.027002	0.000006
N	1.994755	-0.971284	-0.000144
N	-2.246356	-0.701154	-0.000007
N	-1.111216	-0.307233	0.000236
N	-0.711435	0.975156	-0.000411

Tetrahydrofuran in THF

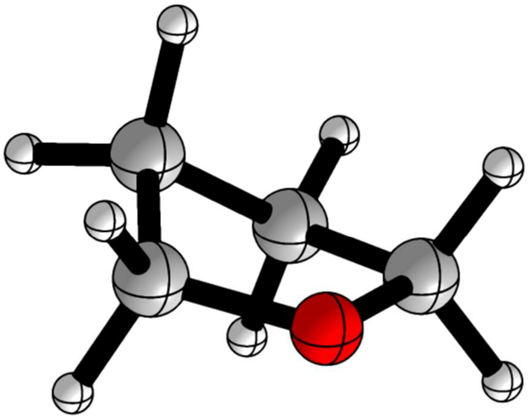

M06-2X/6-31G(d)-IEFPCM(THF) Energy	-232.339616
M06-2X/6-311G(d,p)-IEFPCM(THF) Energy	-232.405522
M06-2X/6-311G(d,p)-IEFPCM(THF) Free Energy (Quasiharmonic)	-232.313446
Number of Imaginary Frequencies	0

M06-2X/6-31G(d)-IEFPCM(THF) Molecular Geometry in Cartesian Coordinates

C	1.160344	-0.426258	0.136872
O	-0.000209	-1.245574	-0.000040
C	-1.160494	-0.425880	-0.136851
C	-0.727125	0.990279	0.237438
C	0.727479	0.990038	-0.237454
H	1.512371	-0.465248	1.176889
H	1.949377	-0.822978	-0.509093
H	-1.512828	-0.464838	-1.176761
H	-1.949467	-0.822341	0.509356
H	-1.343287	1.758417	-0.234677
H	-0.770345	1.127431	1.322968
H	0.770672	1.127116	-1.322990
H	1.343958	1.757960	0.234599

Table S5. Naming of conformers with associated solvent used for the optimisation and C-H HAT position on Tetrahydrofuran.

**Structure**	**Solvent**	**Position**
Ca1a	Acetonitrile	α-O
Ca1b	Acetonitrile	α-O
Ca1c	Acetonitrile	α-O
Ca1d	Acetonitrile	α-O
Ca2a	Acetonitrile	β-O
Ca2b	Acetonitrile	β-O
Ca2c	Acetonitrile	β-O
Ca2d	Acetonitrile	β-O
Cb1a	THF	α-O
Cb1b	THF	α-O
Cb1c	THF	α-O
Cb1d	THF	α-O
Cb2a	THF	β-O
Cb2b	THF	β-O
Cb2c	THF	β-O
Cb2d	THF	β-O

Ca1a

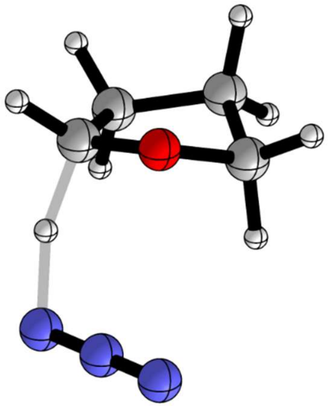

M06-2X/6-31G(d)-IEFPCM(acetonitrile) Energy		-396.398745
M06-2X/6-311G(d,p)-IEFPCM(acetonitrile) Energy		-396.509616
M06-2X/6-311G(d,p)-IEFPCM(acetonitrile) Free Energy (Quasiharmonic)		-396.416396
Number of Imaginary Frequencies	1	-1182.1146

M06-2X/6-31G(d)-IEFPCM(acetonitrile) Molecular Geometry in Cartesian Coordinates

C	-0.909001	1.244147	-0.174972
O	-0.367602	0.548891	0.964535
C	-0.561343	-0.794090	0.776230
C	-1.650443	-1.018484	-0.269174
C	-2.126583	0.415427	-0.565164
H	-1.125847	2.263734	0.142746
H	-0.153768	1.262747	-0.971025
H	0.544917	-1.207587	0.231340
H	-0.586258	-1.329028	1.727111
H	-1.231896	-1.487865	-1.165228
H	-2.444395	-1.665088	0.109825
H	-2.414951	0.562474	-1.606773
H	-2.973058	0.676147	0.075282
N	1.704652	-1.216386	-0.413738
N	2.141434	-0.102309	-0.225127
N	2.555385	0.954601	-0.055574

Ca1b

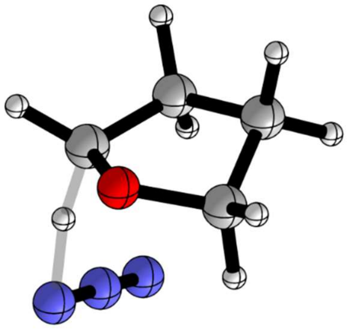

M06-2X/6-31G(d)-IEFPCM(acetonitrile) Energy		-396.396029
M06-2X/6-311G(d,p)-IEFPCM(acetonitrile) Energy		-396.506749
M06-2X/6-311G(d,p)-IEFPCM(acetonitrile) Free Energy (Quasiharmonic)		-396.414294
Number of Imaginary Frequencies	1	-1005.4897

M06-2X/6-31G(d)-IEFPCM(acetonitrile) Molecular Geometry in Cartesian Coordinates

C	1.681784	-0.198455	-1.015511
O	1.464141	-1.155846	0.032075
C	0.604975	-0.587944	0.933247
C	0.707588	0.935199	0.849679
C	1.729278	1.140927	-0.286472
H	2.601932	-0.477455	-1.528343
H	0.838573	-0.251375	-1.716531
H	-0.562645	-0.888870	0.529366
H	0.665657	-1.083120	1.904191
H	-0.259291	1.389258	0.607287
H	1.037035	1.362179	1.799561
H	1.478438	1.978531	-0.938386
H	2.728091	1.304381	0.125449
N	-1.742637	-1.128834	-0.119593
N	-2.316473	-0.064864	-0.183029
N	-2.881272	0.932966	-0.258071

Ca1c

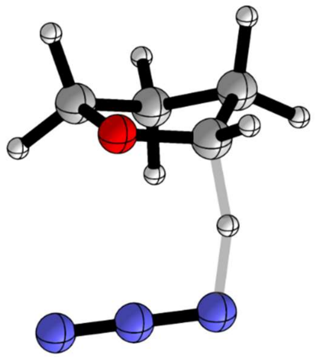

M06-2X/6-31G(d)-IEFPCM(acetonitrile) Energy		-396.400599
M06-2X/6-311G(d,p)-IEFPCM(acetonitrile) Energy		-396.511569
M06-2X/6-311G(d,p)-IEFPCM(acetonitrile) Free Energy (Quasiharmonic)		-396.418195
Number of Imaginary Frequencies	1	-1135.1119

M06-2X/6-31G(d)-IEFPCM(acetonitrile) Molecular Geometry in Cartesian Coordinates

C	-1.003673	-1.326417	0.056980
O	-0.458311	-0.496207	1.109788
C	-0.688618	0.823638	0.830050
C	-1.694524	0.920939	-0.296115
C	-1.377021	-0.364578	-1.069291
H	-0.246916	-2.064327	-0.212281
H	-1.880235	-1.834982	0.470068
H	-0.791811	1.422324	1.736853
H	0.429863	1.248143	0.308760
H	-2.711652	0.896055	0.114063
H	-1.579366	1.830972	-0.887381
H	-2.216143	-0.731567	-1.661507
H	-0.523501	-0.201633	-1.734534
N	1.601967	1.265957	-0.315918
N	1.995666	0.127242	-0.193093
N	2.369404	-0.952745	-0.082716

Ca1d

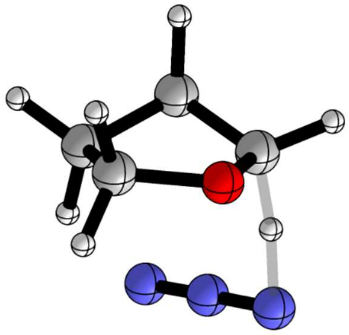

M06-2X/6-31G(d)-IEFPCM(acetonitrile) Energy		-396.397592
M06-2X/6-311G(d,p)-IEFPCM(acetonitrile) Energy		-396.50873
M06-2X/6-311G(d,p)-IEFPCM(acetonitrile) Free Energy (Quasiharmonic)		-396.416201
Number of Imaginary Frequencies	1	-899.6529

M06-2X/6-31G(d)-IEFPCM(acetonitrile) Molecular Geometry in Cartesian Coordinates

C	-1.913401	0.317307	-0.841634
O	-1.598371	-0.998850	-0.341434
C	-0.696905	-0.910065	0.683283
C	-0.654898	0.524862	1.168713
C	-0.956697	1.276534	-0.133717
H	-1.808181	0.301513	-1.927722
H	-2.958681	0.516061	-0.584523
H	-0.818150	-1.727336	1.398447
H	0.456310	-1.141930	0.205473
H	-1.447447	0.682570	1.909948
H	0.298927	0.797001	1.626550
H	-1.406663	2.256190	0.031862
H	-0.042053	1.412412	-0.719160
N	1.737711	-1.211524	-0.301915
N	2.186951	-0.092853	-0.208092
N	2.624525	0.967589	-0.128461

Ca2a

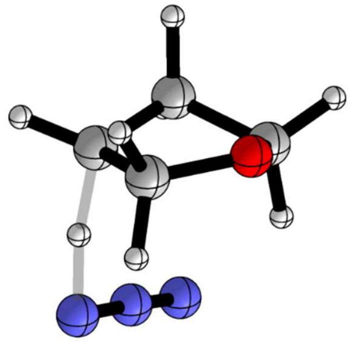

M06-2X/6-31G(d)-IEFPCM(acetonitrile) Energy		-396.38517
M06-2X/6-311G(d,p)-IEFPCM(acetonitrile) Energy		-396.4972
M06-2X/6-311G(d,p)-IEFPCM(acetonitrile) Free Energy (Quasiharmonic)		-396.406009
Number of Imaginary Frequencies	1	-1470.3496

M06-2X/6-31G(d)-IEFPCM(acetonitrile) Molecular Geometry in Cartesian Coordinates

C	0.979638	1.238299	-0.301705
O	1.943231	0.324048	-0.808264
C	1.635006	-0.978294	-0.326604
C	0.680602	-0.787800	0.836972
C	0.625985	0.699865	1.085023
H	1.430812	2.231784	-0.288937
H	0.089957	1.263170	-0.949784
H	1.188362	-1.589297	-1.124363
H	2.566343	-1.462949	-0.011347
H	-0.525319	-1.101621	0.327068
H	0.737928	-1.475413	1.680182
H	-0.335856	1.055858	1.467051
H	1.397604	0.978895	1.814511
N	-2.713561	0.866264	-0.159897
N	-2.175476	-0.141189	-0.190935
N	-1.628545	-1.228680	-0.250652

Ca2b

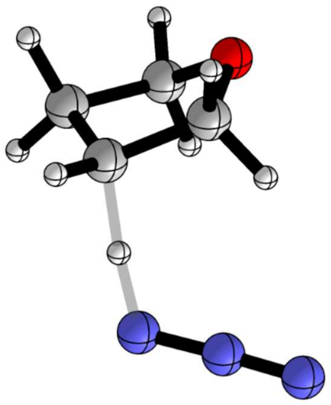

M06-2X/6-31G(d)-IEFPCM(acetonitrile) Energy		-396.384994
M06-2X/6-311G(d,p)-IEFPCM(acetonitrile) Energy		-396.496871
M06-2X/6-311G(d,p)-IEFPCM(acetonitrile) Free Energy (Quasiharmonic)		-396.405898
Number of Imaginary Frequencies	1	-1475.5741

M06-2X/6-31G(d)-IEFPCM(acetonitrile) Molecular Geometry in Cartesian Coordinates

C	1.587019	0.014904	-1.065707
O	1.500256	-1.204181	-0.334706
C	0.723281	-0.979378	0.834010
C	0.635670	0.522759	1.006987
C	1.597522	1.108033	0.001202
H	2.491027	-0.022268	-1.675667
H	0.713369	0.132123	-1.723554
H	-0.268397	-1.448897	0.734775
H	1.223515	-1.454362	1.686791
H	-0.552821	0.893317	0.502641
H	0.583651	0.929507	2.016317
H	1.296002	2.089755	-0.373157
H	2.593891	1.200629	0.452908
N	-2.896431	-0.815623	-0.232688
N	-2.257400	0.128769	-0.164524
N	-1.609490	1.160531	-0.117410

Ca2c

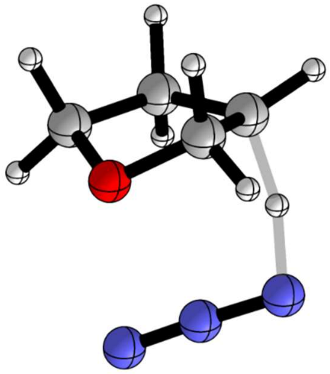

M06-2X/6-31G(d)-IEFPCM(acetonitrile) Energy		-396.38655
M06-2X/6-311G(d,p)IEF-PCM(acetonitrile) Energy		-396.498395
M06-2X/6-311G(d,p)-IEFPCM(acetonitrile) Free Energy (Quasiharmonic)		-396.40635
Number of Imaginary Frequencies	1	-1513.6261

M06-2X/6-31G(d)-IEFPCM(acetonitrile) Molecular Geometry in Cartesian Coordinates

C	1.141939	-1.255500	0.129501
O	0.868231	-0.546664	-1.075508
C	1.301681	0.783705	-0.858180
C	0.847852	1.078210	0.551908
C	0.908453	-0.249611	1.280990
H	0.476813	-2.120147	0.162936
H	2.183386	-1.602543	0.128797
H	2.400809	0.860420	-0.926952
H	0.855642	1.426639	-1.620672
H	1.180688	1.987880	1.049565
H	-0.463322	1.293730	0.355759
H	1.726121	-0.261401	2.008558
H	-0.012929	-0.460533	1.836490
N	-2.194404	-1.081612	-0.122607
N	-1.920068	0.024329	-0.048474
N	-1.670186	1.215631	0.025977

Ca2d

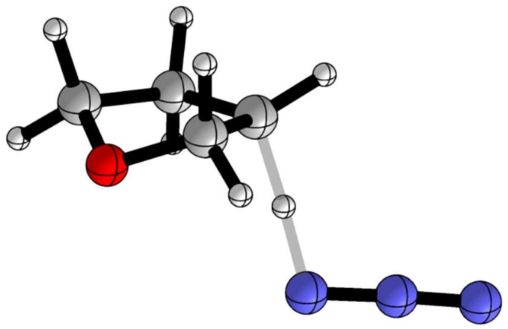

M06-2X/6-31G(d)-IEFPCM(acetonitrile) Energy		-396.384332
M06-2X/6-311G(d,p)-IEFPCM(acetonitrile) Energy		-396.495949
M06-2X/6-311G(d,p)-IEFPCM(acetonitrile) Free Energy (Quasiharmonic)		-396.405601
Number of Imaginary Frequencies	1	-1503.1435

M06-2X/6-31G(d)-IEFPCM(acetonitrile) Molecular Geometry in Cartesian Coordinates

C	-2.405873	0.456191	-0.356803
O	-1.885891	-0.841691	-0.618810
C	-1.070182	-1.159715	0.491536
C	-0.355143	0.133613	0.815767
C	-1.252855	1.244005	0.298090
H	-2.748260	0.878161	-1.302503
H	-3.257345	0.384176	0.334263
H	-1.683854	-1.470258	1.355528
H	-0.408862	-1.986171	0.219082
H	0.125560	0.237821	1.788393
H	0.663739	0.133813	-0.028845
H	-1.598366	1.898072	1.103202
H	-0.727621	1.869714	-0.432187
N	3.639858	-0.049578	0.313580
N	2.644288	0.033118	-0.238963
N	1.605348	0.122693	-0.871473

Cb1a

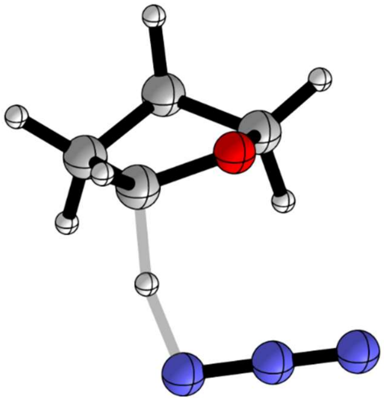

M06-2X/6-31G(d)-IEFPCM(THF) Energy		-396.39747
M06-2X/6-311G(d,p)-IEFPCM(THF) Energy		-396.508306
M06-2X/6-311G(d,p)-IEFPCM(THF) Free Energy (Quasiharmonic)		-396.415044
Number of Imaginary Frequencies	1	-1233.7022

M06-2X/6-31G(d)-IEFPCM(THF) Molecular Geometry in Cartesian Coordinates

C	-0.902297	1.243914	-0.174561
O	-0.371226	0.550134	0.969213
C	-0.564339	-0.792993	0.779951
C	-1.650070	-1.016719	-0.269473
C	-2.120277	0.418127	-0.571149
H	-1.117705	2.265111	0.139078
H	-0.142346	1.258695	-0.966315
H	0.545128	-1.207135	0.235067
H	-0.593601	-1.327446	1.730937
H	-1.228804	-1.489801	-1.162368
H	-2.448421	-1.659683	0.106806
H	-2.402505	0.564097	-1.614695
H	-2.969902	0.682600	0.063695
N	1.695319	-1.216660	-0.415574
N	2.138575	-0.104454	-0.224985
N	2.558943	0.949473	-0.055801

Cb1b

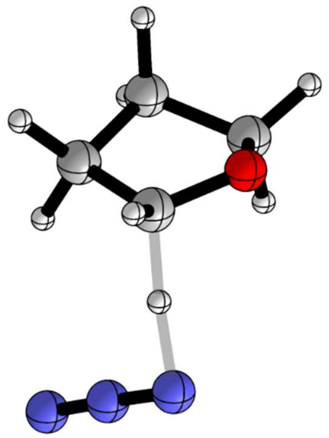

M06-2X/6-31G(d)-IEFPCM(THF) Energy		-396.39472
M06-2X/6-311G(d,p)-IEFPCM(THF) Energy		-396.505417
M06-2X/6-311G(d,p)-IEFPCM(THF) Free Energy (Quasiharmonic)		-396.413111
Number of Imaginary Frequencies	1	-1078.2709

M06-2X/6-31G(d)-IEFPCM(THF) Molecular Geometry in Cartesian Coordinates

C	1.660348	-0.185540	-1.023500
O	1.472542	-1.150335	0.021682
C	0.613572	-0.600243	0.933229
C	0.704858	0.924633	0.867642
C	1.699434	1.151890	-0.289004
H	2.576680	-0.448416	-1.551542
H	0.807562	-0.247985	-1.712107
H	-0.558775	-0.903445	0.524724
H	0.680628	-1.107906	1.897118
H	-0.269944	1.376915	0.655916
H	1.055274	1.341112	1.814926
H	1.421197	1.988037	-0.931746
H	2.703556	1.331360	0.102834
N	-1.728712	-1.132562	-0.125063
N	-2.301423	-0.066700	-0.181614
N	-2.864977	0.931915	-0.251006

Cb1c

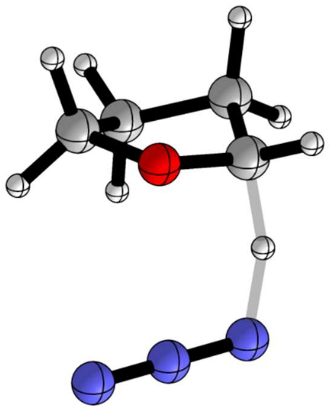

M06-2X/6-31G(d)-IEFPCM(THF) Energy		-396.399318
M06-2X/6-311G(d,p)-IEFPCM(THF) Energy		-396.510258
M06-2X/6-311G(d,p)-IEFPCM(THF) Free Energy (Quasiharmonic)		-396.416894
Number of Imaginary Frequencies	1	-1190.8998

M06-2X/6-31G(d)-IEFPCM(THF) Molecular Geometry in Cartesian Coordinates

C	0.998450	1.326615	0.061619
O	0.466178	0.492102	1.116199
C	0.693608	-0.826704	0.828328
C	1.689634	-0.918654	-0.307271
C	1.363522	0.369828	-1.072120
H	0.237196	2.063337	-0.198178
H	1.877669	1.836965	0.467312
H	0.806167	-1.428723	1.731796
H	-0.431032	-1.250307	0.312695
H	2.711092	-0.895063	0.092470
H	1.568945	-1.825902	-0.901844
H	2.196461	0.740264	-1.671121
H	0.504270	0.207745	-1.730099
N	-1.598047	-1.267403	-0.310268
N	-1.990917	-0.127250	-0.190244
N	-2.364104	0.952990	-0.084625

Cb1d

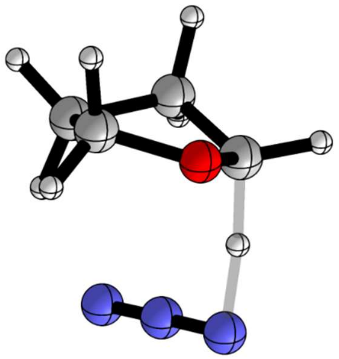

M06-2X/6-31G(d)-IEFPCM(THF) Energy		-396.396291
M06-2X/6-311G(d,p)-IEFPCM(THF) Energy		-396.507417
M06-2X/6-311G(d,p)-IEFPCM(THF) Free Energy (Quasiharmonic)		-396.414912
Number of Imaginary Frequencies	1	-985.3320

M06-2X/6-31G(d)-IEFPCM(THF) Molecular Geometry in Cartesian Coordinates

C	-1.909218	0.324069	-0.841285
O	-1.600620	-0.994828	-0.347141
C	-0.697123	-0.915261	0.676137
C	-0.652314	0.515838	1.172619
C	-0.952911	1.277674	-0.124368
H	-1.798866	0.314174	-1.927032
H	-2.955466	0.524525	-0.588913
H	-0.817648	-1.738514	1.384423
H	0.461995	-1.145608	0.193956
H	-1.443917	0.670511	1.915699
H	0.302475	0.783480	1.631229
H	-1.402040	2.256589	0.048343
H	-0.037269	1.418113	-0.707021
N	1.736375	-1.212254	-0.299487
N	2.183086	-0.091502	-0.208249
N	2.618409	0.969676	-0.131146

Cb2a

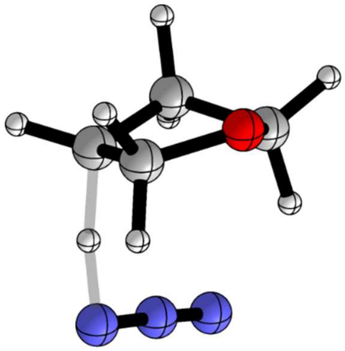

M06-2X/6-31G(d)-IEFPCM(THF) Energy		-396.384015
M06-2X/6-311G(d,p)IEFPCM(THF) Energy		-396.496005
M06-2X/6-311G(d,p)-IEFPCM(THF) Free Energy (Quasiharmonic)		-396.404754
Number of Imaginary Frequencies	1	-1477.8712

M06-2X/6-31G(d)-IEFPCM(THF) Molecular Geometry in Cartesian Coordinates

C	0.986162	1.239106	-0.288072
O	1.949307	0.328774	-0.799670
C	1.623480	-0.978460	-0.346296
C	0.678127	-0.798888	0.827538
C	0.627846	0.686494	1.092359
H	1.439233	2.231499	-0.264293
H	0.098005	1.271999	-0.937999
H	1.160653	-1.563689	-1.154393
H	2.549516	-1.484481	-0.050034
H	-0.535322	-1.108240	0.325109
H	0.740876	-1.494950	1.663522
H	-0.333894	1.041132	1.476257
H	1.398934	0.955925	1.825994
N	-2.697505	0.882037	-0.172584
N	-2.174102	-0.133289	-0.191093
N	-1.643556	-1.230019	-0.236320

Cb2b

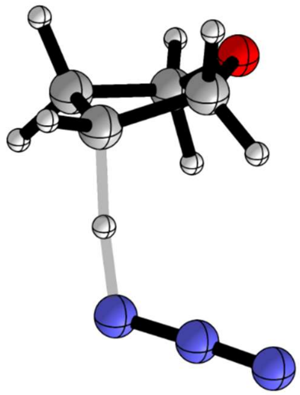

M06-2X/6-31G(d)-IEFPCM(THF) Energy		-396.383795
M06-2X/6-311G(d,p)-IEFPCM(THF) Energy		-396.495625
M06-2X/6-311G(d,p)-IEFPCM(THF) Free Energy (Quasiharmonic)		-396.404597
Number of Imaginary Frequencies	1	-1482.1171

M06-2X/6-31G(d)-IEFPCM(THF) Molecular Geometry in Cartesian Coordinates

C	1.587880	0.019957	-1.064539
O	1.514066	-1.199292	-0.334179
C	0.726234	-0.983605	0.827865
C	0.633463	0.518069	1.008640
C	1.591428	1.112095	0.003873
H	2.490564	-0.009363	-1.676862
H	0.711335	0.130792	-1.719786
H	-0.263950	-1.454025	0.716291
H	1.219620	-1.464116	1.681492
H	-0.557909	0.889215	0.504761
H	0.581439	0.920599	2.019745
H	1.281856	2.092088	-0.368702
H	2.587743	1.211059	0.454393
N	-2.900212	-0.816471	-0.234924
N	-2.259964	0.126641	-0.164172
N	-1.610860	1.158257	-0.114180

Cb2c

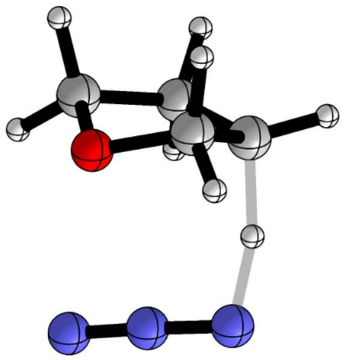

M06-2X/6-31G(d)-IEFPCM(THF) Energy		-396.385237
M06-2X/6-311G(d,p)-IEFPCM(THF) Energy		-396.497049
M06-2X/6-311G(d,p)-IEFPCM(THF) Free Energy (Quasiharmonic)		-396.40497
Number of Imaginary Frequencies	1	-1520.9525

M06-2X/6-31G(d)-IEFPCM(THF) Molecular Geometry in Cartesian Coordinates

C	1.140617	-1.255656	0.129462
O	0.871192	-0.546143	-1.074920
C	1.303983	0.783330	-0.856870
C	0.847429	1.078016	0.552569
C	0.906136	-0.250117	1.281587
H	0.473731	-2.119027	0.160416
H	2.181527	-1.605064	0.131346
H	2.403456	0.860451	-0.924865
H	0.858456	1.425877	-1.620017
H	1.179157	1.987502	1.051437
H	-0.466700	1.293771	0.352558
H	1.722258	-0.263718	2.011070
H	-0.016686	-0.460501	1.834989
N	-2.195258	-1.081051	-0.123550
N	-1.919749	0.024294	-0.049987
N	-1.669810	1.216245	0.025243

Cb2d

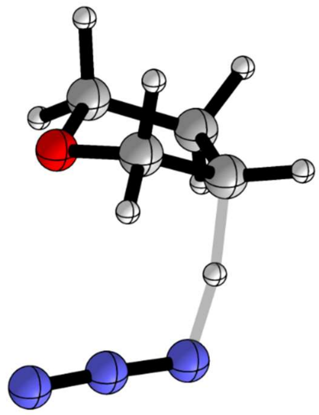

M06-2X/6-31G(d)-IEFPCM(THF) Energy		-396.384506
M06-2X/6-311G(d,p)-IEFPCM(THF) Energy		-396.496181
M06-2X/6-311G(d,p)-IEFPCM(THF) Free Energy (Quasiharmonic)		-396.404591
Number of Imaginary Frequencies	1	-1515.7182

M06-2X/6-31G(d)-IEFPCM(THF) Molecular Geometry in Cartesian Coordinates

C	-1.688374	-0.604464	-0.828463
O	-0.695195	-1.230651	-0.023447
C	-0.656071	-0.497427	1.185405
C	-0.762704	0.941097	0.737153
C	-1.581357	0.909851	-0.539873
H	-1.490375	-0.870393	-1.867485
H	-2.682760	-0.976640	-0.546576
H	-1.509978	-0.755694	1.836576
H	0.267093	-0.747084	1.717499
H	-0.937293	1.720887	1.477190
H	0.479160	1.208731	0.295866
H	-2.562984	1.371849	-0.396967
H	-1.080463	1.448107	-1.351210
N	2.504536	-0.937946	-0.195743
N	2.052601	0.110027	-0.198025
N	1.615750	1.249505	-0.220897

References

1. Bhattacherjee, A.; Sneha, M.; Lewis-Borrell, L.; Amoruso, G.; Oliver, T. A. A.; Tyler, J.; Clark, I. P.; Orr-Ewing, A. J., Singlet and triplet excited state contributions to the activity of dihydrophenazine, phenoxazine and phenothiazine organocatalysts employed in atom transfer radical polymerization. *J. Am. Chem. Soc.* **2021,** *143*, 3613 – 3627
2. Bhattacherjee, A.; Sneha, M.; Lewis-Borrell, L.; Tau, O.; Clark, I. P.; Orr-Ewing, A. J., Picosecond to millisecond tracking of a photocatalytic decarboxylation reaction provides direct mechanistic insights. *Nat. Commun.* **2019,** *10*, 5152.
3. Greetham, G. M.; Donaldson, P. M.; Nation, C.; Sazanovich, I. V.; Clark, I. P.; Shaw, D. J.; Parker, A. W.; Towrie, M., A 100 kHz Time-Resolved Multiple-Probe Femtosecond to Second Infrared Absorption Spectrometer. *Appl. Spectrosc.* **2016,** *70* (4), 645-653.
4. Greetham, G. M.; Sole, D.; Clark, I. P.; Parker, A. W.; Pollard, M. R.; Towrie, M., Time-Resolved Multiple Probe Spectroscopy. *Rev. Sci. Instrumen.* **2012,** *83*, 103107.
5. Koyama, D.; Donaldson, P. M.; Orr-Ewing, A. J., Femtosecond to microsecond observation of the photochemical reaction of 1,2-di(quinolin-2-yl)disulphide with methyl methacrylate. *Phys. Chem. Chem. Phys.* **2017,** *19*, 12981-12991.
6. Sneha, M.; Bhattacherjee, A.; Lewis-Borrell, L.; Clark, I. P.; Orr-Ewing, A. J., Structure-Dependent Electron Transfer Rates for Dihydrophenazine, Phenoxazine, and Phenothiazine Photoredox Catalysts Employed in Atom Transfer Radical Polymerization. *J. Phys. Chem B* **2021,** *125* (28), 7840-7854.
7. Grubb, M. P.; Orr-Ewing, A. J.; Ashfold, M. N. R., KOALA: A program for the processing and decomposition of transient spectra. *Rev. Sci. Instrumen.* **2014,** *85*, 064104.
8. Frisch, M. J.; Trucks, G. W.; Schlegel, H. B.; Scuseria, G. E.; Robb, M. A.; Cheeseman, J. R.; Scalmani, G.; Barone, V.; Mennucci, B.; Petersson, G. A.; Nakatsuji, H.; Caricato, M.; Li, X.; Hratchian, H. P.; Izmaylov, A. F.; Bloino, J.; Zheng, G.; Sonnenberg, J. L.; Hada, M.; Ehara, M.; Toyota, K.; Fukuda, R.; Hasegawa, J.; Ishida, M.; Nakajima, T.; Honda, Y.; Kitao, O.; Nakai, H.; Vreven, T.; Montgomery, J. A.; Peralta, J. E.; Ogliaro, F.; Bearpark, M. J.; Heyd, J. J.; Brothers, E.; Kudin, K. N.; Staroverov, V. N.; Kobayashi, R.; Normand, J.; Raghavachari, K.; Rendell, A.; Burant, J. C.; Iyengar, S. S.; Tomasi, J.; Cossi, M.; Rega, N.; Millam, J. M.; Klene, M.; Knox, J. E.; Cross, J. B.; Bakken, V.; Adamo, C.; Jaramillo, J.; Gomperts, R.; Stratmann, R. E.; Yazyev, O.; Austin, A. J.; Cammi, R.; Pomelli, C.; Ochterski, J. W.; Martin, R. L.; Morokuma, K.; Zakrzewski, V. G.; Voth, G. A.; Salvador, P.; Dannenberg, J. J.; Dapprich, S.; Daniels, A. D.; Farkas, O.; Foresman, J. B.; Ortiz, J. V.; Cioslowski, J.; Fox, D. J. *Gaussian 09*, Gaussian Inc., Wallingford CT, 2009.
9. Becke, A. D., Density-functional thermochemistry. III. The role of exact exchange. *J. Chem. Phys.* **1993,** *98* (7), 5648-5652.
10. Perdew, J. P.; Burke, K.; Ernzerhof, M., Generalized Gradient Approximation Made Simple [Phys. Rev. Lett. 77, 3865 (1996)]. *Phys. Rev. Lett.* **1997,** *78* (7), 1396-1396.
11. Stephens, P. J.; Devlin, F. J.; Chabalowski, C. F.; Frisch, M. J., Ab Initio Calculation of Vibrational Absorption and Circular Dichroism Spectra Using Density Functional Force Fields. *J. Phys. Chem. A* **1994,** *98* (45), 11623-11627.
12. Cancès, E.; Mennucci, B.; Tomasi, J., A new integral equation formalism for the polarizable continuum model: Theoretical background and applications to isotropic and anisotropic dielectrics. *J. Chem. Phys.* **1997,** *107* (8), 3032-3041.
13. Mennucci, B.; Cammi, R.; Tomasi, J., Excited states and solvatochromic shifts within a nonequilibrium solvation approach: A new formulation of the integral equation formalism method at the self-consistent field, configuration interaction, and multiconfiguration self-consistent field level. *J. Chem. Phys.* **1998,** *109* (7), 2798-2807.
14. Frisch, M. J.; Trucks, G. W.; Schlegel, H. B.; Scuseria, G. E.; Robb, M. A.; Cheeseman, J. R.; Scalmani, G.; Barone, V.; Petersson, G. A.; Nakatsuji, H.; Li, X.; Caricato, M.; Marenich, A. V.; Bloino, J.; Janesko, B. G.; Gomperts, R.; Mennucci, B.; Hratchian, H. P.; Ortiz, J. V.; Izmaylov, A.

F.; Sonnenberg, J. L.; Williams-Young, D.; Ding, F.; Lipparini, F.; Egidi, F.; Goings, J.; Peng, B.; Petrone, A.; Henderson, T.; Ranasinghe, D.; Zakrzewski, V. G.; Gao, J.; Rega, N.; Zheng, G.; Liang, W.; Hada, M.; Ehara, M.; Toyota, K.; Fukuda, R.; Hasegawa, J.; Ishida, M.; Nakajima, T.; Honda, Y.; Kitao, O.; Nakai, H.; Vreven, T.; Throssell, K.; Montgomery Jr., J. A.; Peralta, J. E.; Ogliaro, F.; Bearpark, M. J.; Heyd, J. J.; Brothers, E. N.; Kudin, K. N.; Staroverov, V. N.; Keith, T. A.; Kobayashi, R.; Normand, J.; Raghavachari, K.; Rendell, A. P.; Burant, J. C.; Iyengar, S. S.; Tomasi, J.; Cossi, M.; Millam, J. M.; Klene, M.; Adamo, C.; Cammi, R.; Ochterski, J. W.; Martin, R. L.; Morokuma, K.; Farkas, O.; Foresman, J. B.; Fox, D. J. *Gaussian 16 Rev. C.01*, Gaussian Inc. Wallingford, CT, 2016.

15. Simón, L.; Goodman, J. M., How reliable are DFT transition structures? Comparison of GGA, hybrid-meta-GGA and meta-GGA functionals. *Organic & Biomolecular Chemistry* **2011,** *9* (3), 689-700.
16. Funes-Ardoiz, I.; Patoon, R. S. *GoodVibes 2.0.2*, 2016.
17. Legault, C. Y. *CYLView 1.0b*, Université de Sherbrooke: Sherbrooke, Quebec, Canada 2009.
